# Polyphenolic and Chemical Profiles of Honey From the Tara Mountain in Serbia

**DOI:** 10.3389/fnut.2022.941463

**Published:** 2022-06-24

**Authors:** Nebojša Nedić, Milica Nešović, Predrag Radišić, Uroš Gašić, Rada Baošić, Kristina Joksimović, Lato Pezo, Živoslav Tešić, Irena Vovk

**Affiliations:** ^1^Faculty of Agriculture, Institute of Zootechnics, University of Belgrade, Belgrade, Serbia; ^2^Institute of General and Physical Chemistry, Belgrade, Serbia; ^3^BioSense Institute, Research Institute for Information Technologies in Biosystems, University of Novi Sad, Novi Sad, Serbia; ^4^Department of Plant Physiology, Institute for Biological Research “Siniša Stanković”, National Institute of Republic of Serbia, University of Belgrade, Belgrade, Serbia; ^5^Faculty of Chemistry, University of Belgrade, Belgrade, Serbia; ^6^Institute of Chemistry, Technology and Metallurgy, Institute of National Importance for the Republic of Serbia, University of Belgrade, Belgrade, Serbia; ^7^Laboratory for Food Chemistry, National Institute of Chemistry, Ljubljana, Slovenia

**Keywords:** honey, pollen analysis, physicochemical analysis, polyphenols, microbiological analysis, PCA

## Abstract

This study presents a detailed characterization of 27 honey samples from the Tara Mountain region in Serbia using different comprehensive techniques and methods. The types of the honey samples were defined as monofloral (4 samples), honeydew (5 samples) and polyfloral (18 samples) honey based on determined polyphenol content, antioxidant activity, electrical conductivity and melissopalynological analyses. Physicochemical parameters such as pH (4.13–4.94), diastase activity (24.20–41.70 DN), acidity (14.60–29.70 meq/kg), content of 5-(hydroxymethyl)furfural (in range below 5, up to 16.90 mg/kg), sucrose (0.20–3.90 g/100 g), and moisture content (15.01–19.23%) confirmed the required quality of the honey samples. Sensory analysis revealed honey characteristics favorable to consumers. Analyses of 19 phenolic compounds using ultra-high-performance liquid chromatography with a diode-array detection and triple quadrupole mass spectrometry (UHPLC-DAD-MS/MS) revealed six phenolic acids and 13 other compounds from the group of flavonoids and their glycosides. In all the samples the highest content was determined for *p*-coumaric acid, followed by caffeic acid and pinocembrin. Besides total phenolic content and radical scavenging activity, antimicrobial activity was also examined. Most honey samples showed bactericidal activity against *Staphylococcus aureus* and bacteriostatic activity against *Escherichia coli*, while none of the honey samples inhibited the growth of *Candida albicans*. Chemometric analyses were applied for an in-depth study of the results to further evaluate the characteristics of the honey samples studied. Principal component analysis (PCA) was used for assessing the differences in physicochemical parameters, polyphenols content and antioxidant capacity between honey samples. The unrooted cluster tree was used to group the samples based on the melissopalynological analyses.

## Introduction

Honey is a well-known sweet product made by bees. The definition of honey, given by the European Legislation (1), distinguishes honey types such as floral honey and honeydew honey. Floral honey originates from the nectar of blossom plants and can be monofloral or polyfloral. Monofloral honey must contain a minimum of 45% pollen particles of the one plant species that declared its origin (2). Multifloral/polyfloral honey (meadow, blossom) is a product that honeybees produce from the nectar of flowers of different types of honey plants (3). Honeydew honey, is also known as forest honey, is made by the tree excretion or excrement of insects (1). Honeydew is mainly found on fir, pine, spruce, and also on oak, beech, etc.

The Tara Mountain region, belonging to the National Parks of Serbia, is located on the territory of the municipality of Bajina Bašta in the far west of Serbia. The vascular flora of Serbia contains 3662 taxa (4), of which 1000 plant species have been identified in this region (5). Both floral and honeydew honey as well as many other natural products valuable for good health are produced in the Tara Mountain region. With an altitude of 1544 m, Tara Mountain has both deciduous and coniferous forests with profuse meadow plants. Tara Mountain’s ecosystem and biodiversity provide good bee pasture with various possibilities for beekeepers. The meadow pasture on Tara Mountain is most abundant at the end of spring and during the summer, while forest bee pasture prevails at the end of the summer and the beginning of autumn (6). However, meadow or forest bee pastures can also occur at the same time of year, so as to complement each other through the seasons, bees with nectar or honeydew. The environment from which bees collect nectar and pollen determines the composition of honey (7). Due to the high melliferous potential of the diverse plant communities in the Tara Mountain region (6), the analysis of honey samples from this area is highly important.

Given that Serbia (8–10) and its neighboring countries (11–13) have a high potential for honey production, testing of honey is of high importance. Based on published studies the phenolic compounds play a significant role in the bioactive properties of honey (8, 10, 11, 14), and can also serve as potential markers of the botanical and/or geographical origin of honey (10, 12, 15). Determination of physicochemical parameters has proven to be an additional tool for differentiating honey types (8, 9, 16–19) and a good indicator of honey quality (20), which is also dependent on storage conditions that also affect the phenol content (21). The generally accepted melissopalynological analysis, the examination of these parameters provides a more precise assessment of the botanical and geographical origins of the honey (15, 22). A very useful tool for distinguishing honey samples is also statistical analysis (8, 23, 24). Considering taste to be crucial for honey consumption, sensory analysis is of great importance. The parameters determined by sensory analyses can be dependent on the chemical constituents and their content in the honey, which was shown for the honey color and phenolic content (11), as well as for the botanical origin of honey (25). The color of the honey is related to the composition of phenolic compounds (14, 16), with total flavonoid content showing the highest influence (25). A correlation between phenolic composition and antioxidant properties of honey (16) was also noted. Antimicrobial activity against *Staphylococcus aureus* was observed for many types of honey (14). It was found that the antibacterial and antioxidant properties of honey mostly depend on the geographical origin and to a lesser extent on the type of honey (14).

The aim of this study was to provide a detailed characterization of honey samples from Tara Mountain located in the municipality of Bajina Bašta in Serbia, which as a protected area and national park has a very diverse plant flora that can provide good bee pastures. In order to confirm the floral origin of honey samples, melissopalynological analysis was performed. Following international requirements, physicochemical parameters, which contribute to the confirmation of the origin of honey, as well as the properties related to the quality of honey, were determined. In addition to phenolic analysis, honey samples were also analyzed for antioxidant and antimicrobial activity. To further differentiate honey samples a chemometric analysis of all results was performed in order to obtain additional data for the assessment of the botanical and geographical origin of honey.

## Materials and Methods

### Honey Samples

**FIGURE 1:**
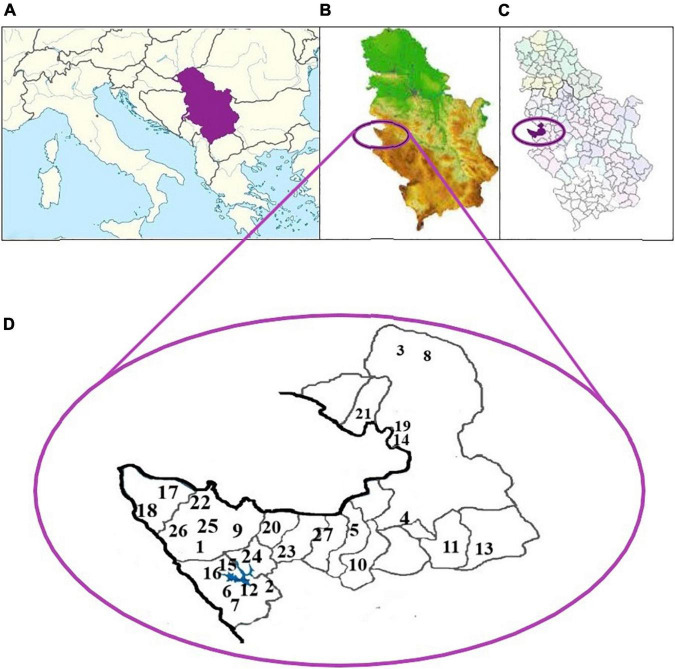
**(A)** Regional map of Europe with Serbia highlighted; **(B)** geographical map of Serbia with the Tara Mountain region highlighted in the Western part of Serbia; **(C)** municipal map of Serbia with the Tara Mountain region highlighted in the Western part of Serbia; **(D)** twenty-seven locations of honey sampled in the Tara Mountain region.

Honey samples were collected from different locations in the Tara Mountain region in Western Serbia in 2019 (Figure 1). Prior to the collection of honey, beehives were placed on meadow pastures at different locations (Table 1).

**TABLE 1:** Data on 27 honey samples collected in 2019 from the Tara Mountain region in Serbia.

No.	Month of bee pasture	Location of honeycombs on meadow pasture
1	June-July	Rastište – Kremići; Zaovine – Nikolići
2	May-August	Zaovine – Gornji Graovac
3	June-August	Pašina ravan – Vinèina voda
4	June-August	Pilica – Pridoli, village Drajići
5	Until July	Raèa
6	June-August	Zaovine, Grkovići
7	June-August	Zaovine
8	Until August	Pašina ravan
9	Until July	Rastište
10	Until July	Raèa – Mala Reka
11	Until July	Zlodol
12	June-August	Zaovine, Lazića Brana
13	May-July	Zaglavak (toward Kadinjaèa)
14	June-July	Ragaèica
15	May-August	Zaovine, Bjeluša
16	Until July	Zaovine, Bjeluša
17	Until August	Jagoštica
18	May-July	Jagoštica
19	June-August	Rogaèica
20	Until July	Perućac
21	Until July	Okletac
22	Until July	Rastište
23	June-August	Beserovina
24	Until July	Zaovine, Konjska reka
25	Until August	Rastište – Križevac
26	June-July	Rastište
27	June-July	Beserovina – Zaugline – Sokolina

### Sensory Analysis

Sensory properties of the 27 honey samples were evaluated by four independent experienced evaluators. Each evaluator was asked to evaluate parameters such as color, odor and flavor attributes, as well as overall appearance and consistency. The intensity of each parameter was described by evaluators using several assigned descriptors (see Table 2).

**TABLE 2:** Sensory analyses of 27 honey samples from the Tara Mountain region in Serbia.

No.	Type of color	Odor attributes		Flavor attributes				Consistency
		Intensity	Odor	Sweetness	Sourness	Other	Persistence of background taste	
1	Light brown	Medium	Fruity	Medium	Medium	**−**	Medium	Crystallized (large, sharp crystals)
2	Dark yellow	Low	**−**	Medium	Medium	**−**	Medium	Crystallized (large, sharp crystals)
3	Amber with reddish tone	Medium	Herbal, woody	**−**	Intensely	**−**	Medium	Thick liquid
4	Amber with reddish tone	Medium	Herbal	Medium	Intensely	**−**	Medium	Thick liquid
5	Amber yellow	Medium	Herbal	Medium	Medium	**−**	Medium	Partially crystallized (fine crystals)
6	Brown-yellow	Medium	Herbal	**−**	Intensely	**−**	Medium	Crystallized (medium size, sharp crystals)
7	Amber with reddish tone	Medium	Herbal, fruity	Medium	Medium	**−**	Medium	Thick liquid
8	Light brown (milk-caramel)	Medium	Herbal (herbaceous plants)	Medium	Lesser extent	**−**	Not	Crystallized (large, sharp crystals)
9	Amber yellow	High	Sour, fruity	**−**	Extremely	**−**	Medium	Thick liquid
10	Brown-cream	Medium	Herbal (on straw)	Sweet	**−**	**−**	Very	Crystallized (medium size crystals)
11	Amber yellow	Very high	Sour, fruity	**−**	Very	**−**	Persistent (background taste)	Thick liquid
12	Brown-cream	Medium	Herbal (on grass)	Sweet	Slightly	**−**	Medium firm	Crystallized (medium size crystals)
13	Light brown	Medium	Herbal (on grass)	Medium	Very	Astringent	Creamy, persistent	Crystallized (large, sharp crystals)
14	Dark yellow	Medium	Herbal (on grass)	Very candy	**−**	**−**	Medium	Thick liquid
15	Amber with red tone	Very high	Fruity	Medium	**−**	**−**	Medium	Thick liquid
16	Dark amber with reddish tone	Medium	Fruity (on wax)	**−**	Intensely	Astringent	Moderately	Thick liquid
17	Amber with reddish tone	Medium	Herbal (on grass)	**−**	Medium	Slightly bitter	Medium	Thick liquid
18	Amber with reddish tone	Medium	Herbal, fruity	Medium	Medium	**−**	Medium	Thick liquid
19	Dark amber	Medium	Herbal	Toasted sugar	Medium	**−**	Medium	Thick liquid
20	Amber	Medium	Herbal (on grass), chemical	**−**	Slightly	Slightly bitter	Medium firm	Thick liquid
21	Amber	Very high	On flowers (lilac)	Medium	Medium	**−**	Medium	Thick liquid
22	Dark brown-yellow	Medium	**−**	Medium	Medium	**−**	Medium	Thick liquid
23	Amber with reddish tone	Medium	Herbal (on grass)	Medium	Medium	**−**	Medium	Thick liquid
24	Dark amber	Medium	On wax	Medium	Medium	**−**	Medium	Thick liquid
25	Dark brown-yellow	Medium	Herbal (on grass)	Medium	Medium	**−**	Medium	Crystallized (small, sharp crystals)
26	Amber with reddish tone	Medium	Herbal (on grass)	Medium	Intensely	**−**	Medium	Thick liquid
27	Brown-yellowish	Medium	Fruity	Medium	Medium	**−**	Medium	Crystallized (fine crystals)

### Chemicals

Ultra-pure water (≤0.055 μS/cm) was obtained by using the water purification system TKA Micro Pure (Thermo Fisher TKA, Niederelbert, Germany). Methanol, hydrochloric acid and acetonitrile were supplied by Merck (Darmstadt, Germany). The Folin-Ciocalteu’s reagent, gallic acid, 2,2-diphenyl-1-picrylhydrazyl (DPPH) and Trolox standard (6-hydroxy-2,5,7,8-tetramethylchrom-2-carboxylic acid), as well as phenolic standards (protocatechuic acid, syringic acid, chlorogenic acid, caffeic acid aesculetin, rutin, *p*-coumaric acid, quercetin 3-*O*-glucoside, ellagic acid, quercetin 3-*O*-rhamnoside, eriodictyol, luteolin, quercetin, naringenin, kaempferol, hispidulin, isorhamnetin, pinocembrin and galangin) were purchased from Sigma-Aldrich (Steinheim, Germany). Miller-Hinton agar and Sabouraud dextrose agar were purchased from Torlak (Belgrade, Serbia). Polytetrafluoroethylene (PTFE) membrane syringe filters (13 mm; 0.45 μm) were obtained from Supelco (Bellefonte, PA, United States) and cartridges for solid phase extraction (SPE; Strata C18-E column; 55 μm, 70 Å; 500 mg/3mL) were purchased from Phenomenex (Torans, CA, United States).

### Sample Extraction

A methanol solution (5 mL of 70% methanol_(aq)_, acidified with 0.1% HCl to pH 2) was added to each honey sample (5 g). The extracts were prepared using ultrasound assisted extraction (1 h) and were filtered through 0.45 μm polytetrafluoroethylene (PTFE) membrane syringe filters. The supernatants assigned as solutions A1–A27 were used for antioxidant activity. Concentration and isolation of polyphenols from the solutions A1–A27 were performed using the SPE Strata C18-E cartridges and acetonitrile as described in our previous study (13). The obtained solutions for polyphenol analyses (assigned as P1–P27) were stored in glass storage vials at 4°C and were used undiluted for UHPLC–DAD-MS/MS analyses of polyphenols.

### Melissopalynology Analyses

The type of pollen particles and the relative frequency of each pollen type in the honey samples were determined by procedures described in the literature (2, 26).

### Determination of Physicochemical Parameters

Physicochemical parameters (pH, diastase activity, electrical conductivity, acidity, 5-(hydroxymethyl)furfural (5-HMF), glucose and fructose content, moisture content and water insoluble impurities) of the 27 honey samples were determined according to the regulations and standards for honey analysis (20).

### Total Phenolic Content and Radical Scavenging Activity

The antioxidant capacity of solutions (A1–A27) was measured by determining total phenolic content (TPC) and radical scavenging activity (RSA) parameters following the method described in our previous study (8). The yellow color of Folin-Ciocalteu’s reagent (used for TPC parameter) turns blue upon reaction with polyphenols. The dark purple color of the methanol solution of DPPH (used for RSA parameter) turns yellow in the presence of antioxidants in the extracts. Monitoring of the reactions was performed using an UV-Vis spectrophotometer (Thermo Scientific Evolution 600; Thermo Fisher Scientific Inc.) at 765 and 517 nm for TPC and RSA, respectively.

### Quantification of Polyphenols

Determination of phenolic compounds in solutions P1–P27 was performed on an ultra-high-performance liquid chromatography system (UHPLC) with a diode-array detector (DAD) connected to a triple quadrupole mass spectrometer (TSQ Quantum Access Max, Thermo Fisher Scientific, Basel, Germany). Heated electrospray ionization (HESI) and multiple mass spectrometric scanning (SRM) were applied for analyses. The system was supported with the Xcalibur software version 2.2. Separation of phenolic compounds was performed on a reversed phase Syncronis C18 analytical column (100 mm × 2.1 mm; 1.7 μm) using a polar mobile phase consisting of (A) water with 0.1% formic acid and (B) acetonitrile. A flow rate was 0.3 mL/min, injection volume 5 μL and linear gradient program was as follows: 0–1 min 5% B; 1–9.9 min from 5 to 95% B; 9.9–10 min from 95 to 5% of B; 10–13 min 5% of B (13). Quantifications of phenolic compounds were performed by recording mass chromatograms of molecular ions and selecting the two most intense fragments from MS^2^ fragmentation. Calibration curves were made using methanol solutions of the mixture of phenolic standards (initial concentration of 1000 mg/L). In comparison with the mass spectra obtained for phenolic standards, as well as the integration of the peaks obtained for P1–P27 solutions, the concentrations of observed phenolic compounds were calculated.

### Examination of Antimicrobial Activity

Antimicrobial activity was determined by the diffusion of a 50% honey solution (prepared with ultrapure water) through an agar medium. Miller-Hinton medium was used for aerobic bacteria *Escherichia coli* (ATCC 25922) and *Staphylococcus aureus* (ATCC 25923). Sabouraud-dextrose medium was used for fungus *Candida albicans* (ATCC 24433). Both media were prepared according to the manufacturer’s instructions. The suspensions of microorganisms were made in a physiological solution in the concentration range of 10^4^–10^6^ CFU/mL, then 100 μL of suspensions were mixed with 20 mL of their respective media (Miller-Hinton agar/Sabouraud dextrose agar). The resulting thickness in the Petri dish was approximately 4–5 mm. The aqueous honey solution (100 μL) was then added into a hole-tank (diameter 13 mm) made in the middle of the Petri dish. Diffusion for 2 h at 4°C was followed by incubation at 37°C for bacteria and 28°C for fungus for 18–24 h.

### Chemometric Analyses

The results of principal component analysis (PCA) of 27 samples from the Tara Mountain region in Serbia are presented in biplots according to research variables such as: physicochemical parameters, quantification of polyphenols, antioxidant capacity and antibacterial activity of honey samples. The PCA analysis was introduced to classify the samples by decomposing the original data matrices into loadings and score matrices. Honey samples were taken as variables (column of the input matrix) and the research variables as statistical cases (rows of the matrix). The Pearson’s correlation between obtained variable sets were also investigated in order to assess and explain the linear relationship between observed variables. This data was analyzed using StatSoft Statistica 12 (StatSoft Inc., Tulsa, OK, United States). The melissopalynology analyses data was assessed using R software, 4.0.2 (64-bit version).

## Results and Discussion

### Sensory Analyses

The results of the sensory analyses of 27 honey samples are presented in Table 2. The color descriptions of the honey samples included brown, yellow and amber with different shades and tones. Most of the samples (16 of 27) were amber colored. Honey color could affect consumer preference and could also be connected to the phenolic composition (11). Odor attributes included properties such as intensity of odor (low, medium, high and very high) and an odor (reminiscent of fruit, herbal, wood, grass, straw, wax or flowers). Flavor attributes were defined by type of flavor (sweetness, sourness or other) and persistence of background taste. It was observed that most samples (Nos. 1, 4, 5, 7, 8, 13, 18, 22–27; see Table 2) had medium intensity of odor as well as medium sweetness.

Such characteristics are usually appreciated by consumers. Some samples with intensive sour flavor had medium intensity of sweetness (Nos. 4, 13, and 26). “Very” and “extremely” sour flavors were noticed for samples Nos. 9, 11, and 16 (Table 2) and their sourness could not be mitigated by other observed properties.

The consistency of the honey samples was mainly thick liquid (17 samples). This consistency was also observed in four of five honeydew honey samples (Nos. 16, 20, 22, 23, and 25; section “Physicochemical parameters”). As seen in Table 2, one honey sample was partially crystallized with fine crystals (No. 5) and nine samples were crystallized with different types and sizes of crystals (Nos. 1, 2, 5, 6, 8, 10, 12, 13, 25, and 27; see Table 2). In addition, the crystallized samples did not have the lowest moisture content (Table 3). Crystallization of honey, noted for 10 honey samples (Table 2), is usually less accepted by consumers in Serbia, although it indicates a good quality of honey due to less water content. When it comes to honey consumption, the sensory attributes of honey predominate as more important than any other properties. By observing the parameters of sensory analyses (Table 2), there was no priority for the selection of honey samples. In addition, the sensory analyses showed the acceptability of the examined honey samples despite the fact that the evaluators did not assign numerical values.

**TABLE 3:** Physicochemical parameters with the descriptive analysis of analyzed honey samples from the Tara Mountain region in Serbia.

Type of honey	Sample No.	pH	Diastase activity (DN)	Electrical conductivity (mS/cm)	Acidity (meq/kg)	5-HMF (mg/kg)	Sucrose (g/100 g)	Sugar (Glu + Fru) (g/100 g)	Moisture content (%)	Water insoluble impurities (g/100 g)
Polyfloral honey	1	4.63	33.20	0.65	29.60	<5.00	2.80	68.00	19.23	<0.01
Monofloral honey	2	4.54	34.10	0.44	25.80	<5.00	0.60	67.90	16.31	<0.01
Polyfloral honey	3	4.21	38.60	0.33	29.70	9.30	0.30	72.30	17.45	<0.01
Polyfloral honey	4	4.27	31.60	0.35	28.90	<5.00	0.40	69.90	18.43	<0.01
Polyfloral honey	5	4.60	33.80	0.57	27.80	<5.00	2.30	68.90	15.33	<0.01
Polyfloral honey	6	4.13	33.80	0.29	28.00	5.80	0.70	73.40	17.95	<0.01
Polyfloral honey	7	4.36	29.20	0.36	17.00	11.90	2.60	68.80	16.44	<0.01
Polyfloral honey	8	4.13	33.60	0.27	27.60	<5.00	0.50	70.60	17.91	<0.01
Polyfloral honey	9	4.67	34.20	0.62	18.20	<5.00	1.80	68.80	16.40	<0.01
Polyfloral honey	10	4.68	32.00	0.61	18.60	<5.00	3.30	74.40	16.76	<0.01
Polyfloral honey	11	4.32	29.10	0.34	17.90	7.50	2.20	72.60	15.71	<0.01
Polyfloral honey	12	4.33	36.60	0.42	25.10	5.00	2.60	72.10	18.40	<0.01
Monofloral honey	13	4.38	26.70	0.40	16.80	6.50	1.10	72.70	17.04	<0.01
Polyfloral honey	14	4.18	29.60	0.27	14.60	8.70	1.30	73.10	17.98	<0.01
Polyfloral honey	15	4.76	32.20	0.75	18.90	<5.00	2.90	70.90	16.43	<0.01
Honeydew honey	16	4.90	29.90	0.89	21.70	<5.00	3.50	68.20	18.44	<0.01
Monofloral honey	17	4.69	41.70	0.61	18.90	<5.00	3.50	72.80	16.12	<0.01
Polyfloral honey	18	4.36	33.30	0.37	20.40	7.70	2.30	68.40	16.92	<0.01
Polyfloral honey	19	4.16	30.20	0.40	29.60	14.90	0.50	76.40	17.09	<0.01
Honeydew honey	20	4.86	30.20	0.84	20.80	<5.00	1.00	71.90	17.32	<0.01
Monofloral honey	21	4.75	24.20	0.36	18.20	16.90	3.90	76.90	15.01	<0.01
Honeydew honey	22	4.94	32.10	1.06	21.30	<5.00	0.20	66.10	16.69	<0.01
Honeydew honey	23	4.89	32.00	0.92	22.10	<5.00	2.50	66.30	16.45	<0.01
Polyfloral honey	24	4.70	29.10	0.74	17.50	<5.00	2.30	72.80	16.33	<0.01
Honeydew honey	25	4.88	37.90	1.16	20.20	<5.00	3.40	67.90	16.11	<0.01
Polyfloral honey	26	4.82	31.50	0.70	19.80	<5.00	3.70	75.50	16.08	<0.01
Polyfloral honey	27	4.64	31.00	0.58	22.80	<5.00	1.80	74.30	17.33	<0.01
Monofloral honey (Nos. 2, 13, 17, and 21)	Max	4.75	41.70	0.61	25.80	16.90	3.90	76.90	17.04	/
	Min	4.38	24.20	0.36	16.80	6.50	0.60	67.90	15.01	/
	Average	4.59	31.68	0.45	19.93	/	2.28	72.58	16.12	/
	SD	0.17	7.90	0.11	4.01	/	1.67	3.68	0.84	/
Polyfloral honey	Max	4.82	38.60	0.75	29.70	14.90	3.70	76.40	19.23	/
	Min	4.13	29.10	0.27	14.60	<5	0.30	68.00	15.33	/
	Average	4.44	32.37	0.48	22.89	/	1.91	71.73	17.12	/
	SD	0.24	2.59	0.17	5.31	/	1.06	2.57	1.04	/
Honeydew honey (Nos. 16, 20, 22, 23, and 25)	Max	4.94	37.90	1.16	22.10	/	3.50	71.90	18.44	/
	Min	4.86	29.90	0.84	20.20	/	0.20	66.10	16.11	/
	Average	4.89	32.42	0.97	21.22	/	2.12	68.08	17.00	/
	SD	0.03	3.22	0.13	0.75	/	1.47	2.33	0.92	/

### Melissopalynology Analyses

Melissopalynological analyses (Table 4) confirmed the presence of *Rubus* pollen particles in honey samples among which nine samples (Nos. 1, 11, 12, 13, 14, 19, 20, 21, and 22) contained more than 20% of *Rubus* pollen particles. Among the nine samples, four samples (Nos. 1, 13, 20, and 21) contained more than 30% and three samples (Nos. 13, 20, and 21) contained more than 40% of *Rubus* pollen particles. Some of the other samples also contained over 40% of pollen particles, but from different plant species like *Filipendula ulmaria* (No. 10), Ericaceae family (Nos. 2 and 23) and *Lotus*-group (No. 17). Additional samples contained more than 20% of pollen particles from species from the Fabaceae family (Nos. 3 and 6), *Hypericum* (No. 7), *Prunus/Malus/Pyrus* (No. 21) or *Filipendula ulmaria* (Nos. 18, 24, and 26) (Table 4). Although *Filipendula ulmaria* is nectarless, its pollen is often collected by bees and is very often found in honey (7, 27).

**TABLE 4:** Melissopalynological analyses of honey samples from the Tara Mountain region in Serbia.

No.	Pollen content					
	>30%	20–30%	10–20%	5–10%	1–5%	<1%
1	*Rubus* 31.08%	−	*Filipendula ulmaria* 18.46%	*Prunus/Malus/Pyrus* 7.69%, Poaceae 6.15%	Apiaceae A-type 3.69%, Fabaceae 3.69%, *Rumex* 3.08%, Ericaceae 2.77%, Cistaceae 2.46%, *Plantago* 2.46%, Cyperaceae 2.15%, *Robinia pseudoacacia* 2.15%, *Fagus* 2.15%, Asteraceae J-form (*Centaurea jacea*) 1.23%, *Hypericum* 1.23%, *Sanguisorba minor* 1.23%	*Clematis* 0.92%, Gallium 0.92%, Brassicaceae 0.62%, *Trifolium repens* -group 0.62%, *Juglans* 0.62%, *Tilia* 0.62%, *Urtica* 0.62%, Others 0.03%
2	Ericaceae 59.55%	−	*Rubus* 18.91%	*Salix* 6.55%	Lamiaceae M-form (*Origanum, Thymus, Mentha, Melissa*) 4.12%, *Prunus/Malus/Pyrus* 3.18%, *Filipendula ulmaria* 1.87%, *Viola* type 1.31%, Fabaceae 1.12%	Others 0.03%
3	−	Fabaceae 23.23%	*Rubus* 16.16%	*Filipendula ulmaria* 9.90%, *Hypericum* 7.27%	*Lotus*-group 4.44%, *Prunus/Malus/Pyrus* 4.44%, Undefined 2 3.23%, Lamiaceae S-form (*Salvia*) 3.03%, *Rumex* 3.03%, Asteraceae J-form (*Centaurea jacea*) 2.42%, *Robinia pseudoacacia* 2.42%, Tetracolpate 2.42%, Unidentified 3.22%, Poaceae 2.02%, *Salix* 1.62%, Trifolium 1.41%, *Echium* 1.21%, *Plantago* 1.21%, Apiaceae H-type 1.01%, *Vicia* -type 1.01%, *Geranium* 1.01%	Asteraceae – *Senecio* type (*Senecio, Solidago*) 0.81%, *Sanguisorba minor* 0.81%, *Cornus sanguinea* 0.61%, *Knautia arvensis* 0.61%, *Trifolium repens* – group 0.61%, Others 0.02%
4	−	−	*Rubus* 15.66%, *Plantago* 13.19%, *Robinia pseudoacacia* 10.71%	Fabaceae 9.89%, *Filipendula ulmaria* 6.59%, Asteraceae – *Senecio* type (*Senecio, Solidago*) 5.77%	*Rumex* 4.67%, *Lotus* – group 3.85%, Poaceae 3.30%, Asteraceae T-form (*Taraxacum, Cichorium*) 3.02%, *Amorpha fruticosa* 2.75%, *Hypericum* 2.47%, *Clematis* 2.47%, Apiaceae H-type 2.20%, Lamiaceae M-form (*Origanum, Thymus, Mentha, Melissa*) 2.20%, *Sanguisorba minor* 1.92%, *Prunus/Malus/Pyrus* 1.92%, Lamiaceae S-form (*Salvia*) 1.65%, *Vicia*-type 1.37%, *Galium* 1.10%	*Salix* 0.55%, Tetracolpate 0.55%, Others 0.02%
5	Ericaceae 38.21%	−	*Rubus* 11.41%	Fabaceae 7.69%, *Robinia pseudoacacia* 6.95%, *Filipendula ulmaria* 6.70%	*Prunus/Malus/Pyrus* 4.96%, *Salix* 3.47%, *Clematis* 2.98%, *Plantago* 2.48%, *Lotus* -group 1.99%, *Rumex* 1.74%, *Quercus* 1.49%, Pinaceae 1.24%, *Sanguisorba minor* 1.24%	Asteraceae – *Senecio* type (*Senecio, Solidago*) 0.99%, *Hypericum* 0.99%, Lamiaceae S-form (*Salvia*) 0.99%, Apiaceae H-type 0.74%, Poaceae 0.74%, Tetracolpate 0.74%, Others 0.02%
6	−	Fabaceae 24.53%	*Rubus* 13.21%, *Clematis* 10.38%	*Filipendula ulmaria* 8.49%, Lamiaceae S-form (*Salvia*) 6.60%	*Vicia*-type 4.72%, Unidentified 2 3.77%, *Echium* 2.83%, *Lotus* – group 2.83%, *Prunus/Malus/Pyrus* 2.83%, Unidentified 4 2.83%, Asteraceae J-form (*Centaurea jacea*) 1.89%, *Campanula* 1.89%, *Hypericum* 1.89%, *Epilobium* 1.89%	*Allium Muscari* type 0.94%, Apiaceae H-type 0.94%, Asteraceae T-form (*Taraxacum, Cichorium*) 0.94%, Asteraceae S-form (*Carduus/Cirsium/Serratula*) 0.94%, Ericaceae 0.94%, *Trifolium* 0.94%, *Fagus* 0.94%, *Plantago* 0.94%, *Sanguisorba minor* 0.94%, Unidentified 14 0.94%
7	−	*Hypericum* 22.97%	−	*Rumex* 8.11%, *Lotus*-group 7.84%, *Rubus* 5.68%, Unidentified 55.68%	Asteraceae J-form (*Centaurea jacea*) 4.59%, Apiaceae À-type 4.05%, Fabaceae 4.05%, Lamiaceae S-form (*Salvia*) 4.05%, *Plantago* 3.78%, *Prunus/Malus/Pyrus* 3.78%, Poaceae 2.70%, *Amorpha fruticosa* 2.16%, *Robinia pseudoacacia* 1.89%, Asteraceae S- form (*Carduus/Cirsium/Serratula*) 1.62%, Lamiaceae M-form (*Origanum, Thymus, Mentha, Melissa*) 1.62%, *Filipendula ulmaria* 1.62%, Unidentified 2 1.62%, Asteraceae T-form (*Taraxacum, Cichorium*) 1.35%	*Artemisia* 0.81%, Asteraceae-*Senecio* type (*Senecio, Solidago*) 0.81%, Brasicaceae 0.81%, Apiaceae H-type 0.54%, Asteraceae- *Senecio* type (*Ambrosia* form) 0.54%, *Trifolium* 0.54%, *Gleditshia* 0.54%, *Fraxinus ornus* 0.54%, *Thalictrum* 0.54%, *Clematis* 0.54%, *Salix* 0.54%, *Urtica* 0.54%, Others 0.04%
8	−	−	*Filipendula ulmaria* 16.35%, *Rubus* 14.26%, Asteraceae J – form (*Centaurea jacea*) 14.05%	Fabaceae 8.81%, *Hypericum* 6.50%, Lamiaceae M – form (*Origanum, Thymus, Mentha, Melissa*) 6.50%	*Trifolium* 4.61%, *Robinia pseudoacacia* 3.77%, *Prunus/Malus/Pyrus* 3.35%, Lamiaceae L – form (*Lamium*) 1.89%, Unknown 5 1.89%, Apiaceae H-type 1.68%, *Lotus* – group 1.68%, *Plantago* 1.68%, Poaceae 1.68%, *Amorpha fruticosa* 1.26%, Asteraceae – *Senecio* type (*Senecio, Solidago*) 1.05%, *Echium* 1.05%, Tetracolpate 1.05%	Brassicaceae 0.84%, *Lythrum* 0.84%, *Salix* 0.84%, Unknown 2 0.84%, Asteraceae A – form (*Achillea*) 0.63%, Asteraceae S – form (*Carduus/Cirsium/Serratula*) 0.63%, *Rumex* 0.63%, Others 0.02%
9	*Clematis* 33.27%	−	*Rubus* 14.08%, *Filipendula ulmaria* 10.05%	Asteraceae J-form (*Centaurea jacea*) 8.41%, *Lotus*-group 7.68%	Rhamnaceae 4.02%, *Tilia* 3.11%, Fabaceae 2.93%, *Prunus/Malus/Pyrus* 2.01%, Lamiaceae S-form (*Salvia*) 1.83%, Poaceae 1.65%, *Echium* 1.28%, Asteraceae S-form (*Carduus/Cirsium/Serratula*) 1.10%, *Rumex* 1.10%	*Trifolium repens* – group 0.91%, *Galium* 0.91%, *Amorpha fruticosa* 0.73%, Apiaceae A-type 0.55%, Cistaceae 0.55%, *Vicia* -type 0.55%, Others 0.03%
10	*Filipendula ulmaria* 54.87%	−	*Rubus* 19.83%	−	*Prunus/Malus/Pyrus* 4.27%, Fabaceae 1.88%, *Rumex* 1.88%, *Robinia pseudoacacia* 1.54%, Lamiaceae L-form (*Lamium*) 1.37%, Zea Mays 1.20%, Tetracolpate 1.20%, *Vicia* -type 1.03%	Asteraceae T-form (Taraxum, Cichorium) 0.68%, Asteraceae- *Senecio* type (*Senecio, Solidago*) 0.51%, *Echium* 0.51%, Brassicaceae 0.51%, *Plantago* 0.51%, Unidentified 13 0.51%, Others 0.03%
11	−	*Rubus* 24.06%, *Filipendula ulmaria* 23.66%	−	Apiaceae H-type 7.75%, Fabaceae 7.75%, *Prunus/Malus/Pyrus* 6.36%	Poaceae 4.17%, *Rumex* 3.78%, *Trifolium repens* – group 2.98%, Asteraceae- *Senecio* type (*Senecio, Solidago*) 2.58%, *Hypericum* 1.79%, Brasicaceae 1.39%, *Robinia pseudoacacia* 1.19%, *Vicia* -type 1.19%	Asteraceae- *Senecio* type (*Erigeron* form) 0.99%, Cistaceae 0.99%, *Plantago* 0.99%, Asteraceae J-form (*Centaurea jacea*) 0.80%, Asteraceae T-form (*Taraxacum, Cichorium*) 0.80%, Unknown 5 0.80%, *Sambucus* 0.60%, *Clematis* 0.60%, *Sanguisorba minor* 0.60%, *Galium* 0.60%, Others 0.04%
12	−	*Rubus* 28.07%	*Hypericum* 19.30%	*Filipendula ulmaria* 8.55%, Fabaceae 5.70%	Asteraceae J-form (*Centaurea jacea*) 4.82%, Cistaceae 3.73%, *Amorpha fruticosa* 3.29%, Lamiaceae M-form (*Origanum, Thymus, Mentha, Melissa*) 2.19%, Tetracolpate 2.19%, *Castanea sativa* 1.98%, Lamiaceae S-form (*Salvia*) 1.97%, *Prunus/Malus/Pyrus* 1.97%, Asteraceae- *Senecio* type (*Senecio, Solidago*) 1.54%, *Echium* 1.54%, *Plantago* 1.32%, Unidentified 5 1.32%, *Lotus* -group 1.10%, Poaceae 1.10%	Ericaceae 0.88%, Apiaceae H-type 0.66%, Asteraceae T-form (*Taraxacum, Cichorium*) 0.66%, Caryophilaceae 0.66%, *Lythrum* 0.66%, *Clematis* 0.66%, Others 0.04%
13	*Rubus* 47.95%	−	−	Fabaceae 8.81%, *Filipendula ulmaria* 6.85%, *Prunus/Malus/Pyrus* 6.26%, Asteraceae- *Senecio* type (*Senecio*. *Solidago*) 5.68%	*Rumex* 4.31%, *Robinia pseudoacacia* 2.15%, Apiaceae A-type 1.76%, *Plantago* 1.57%, Poaceae 1.37%, Brasicaceae 1.17%, *Sanguisorba minor* 1.17%, Tetracolpate 1.17%, Unidentified 5 1.17%	Asteraceae J-form (*Centaurea jacea*) 0.98%, Apiaceae H-type 0.78%, *Amorpha fruticosa* 0.78%, Asteraceae T-form (*Taraxacum, Cichorium*) 0.59%, *Vicia* -type 0.59%, *Loranthus* 0.59%, *Galium* 0.59%, Others 0.04%
14	−	*Rubus* 26.70%	Fabaceae 11.93%, *Salix* 10.04%	Apiaceae A-type 5.87%, *Prunus/Malus/Pyrus* 5.11%	*Robinia pseudoacacia* 4.73%, *Filipendula ulmaria* 4.73%, Lamiaceae M-form (*Origanum, Thymus, Mentha, Melissa*) 2.84%, *Hypericum* 2.08%, *Saxifraga* 1.89%, *Plantago* 1.70%, Ranunculaceae 1.70%, Tetracolpate 1.70%, Asteraceae- *Senecio* type (*Erigeron* form) 1.52%, *Lotus* -group 1.52%, *Potentilla/Fragaria* 1.52%	*Trifolium repens* – group 0.95%, Poaceae 0.95%, *Clematis* 0.95%, Asteraceae J-form (*Centaurea jacea*) 0.76%, Cistaceae 0.76%, *Urtica* 0.76%, *Acer* 0.57%, Asteraceae- *Senecio* type (*Senecio, Solidago*) 0.57%, *Alnus* 0.57%, *Cornus sanguinea* 0.57%, *Trifolium pratense* – group 0.57%, *Fraxinus ornus* 0.57%, Pinaceae 0.57%, *Rumex* 0.57%, *Tilia* 0.57%, Unidentified 0.57%, Unidentified 12 0.57%, Others 0.03%
15	−	−	*Filipendula ulmaria* 18.72%, *Lotus* – group 14.86%	*Rubus* 7.16%, Apiaceae A-type 6.61%, *Trifolium repens* -group 5.69%, *Hypericum* 5.14%	Asteraceae S-form (*Carduus/Cirsium/Sarratula*) 3.67%, Brasicaceae 3.12%, Cistaceae 2.75%, Lamiaceae S-form (*Salvia*) 2.75%, *Trifolium pratense*-group 2.39%, Apiaceae H-type 1.83%, Poaceae 1.83%, *Clematis* 1.65%, Asteraceae J-form (*Centaurea jacea*) 1.47%, Lamiaceae M-form (*Origanum, Thymus, Mentha, Melissa*) 1.47%, *Potentilla/Fragaria* 1.28%, Asteraceae T-form (*Taraxacum, Cichorium*) 1.10%, *Prunus/Malus/Pyrus* 1.10%, *Tilia* 1.10%	Asteraceae- *Senecio* type (*Senecio, Solidago*) 0.92%, Fabaceae 0.92%, *Rumex* 0.92%, Ranunculaceae 0.92%, *Salix* 0.92%, *Fraxinus ornus* 0.73%, *Plantago* 0.73%, *Urtica* 0.73%, Unidentified 2 0.73%, *Echium* 0.55%, Ericaceae 0.55%, Lamiaceae L form (*Lamium*) 0.55%, Pinaceae 0.55%, Unidentified 0.55%, Others 0.04%
16	−	−	Fabaceae 15.46%, *Filipendula ulmaria* 13.35%, *Hypericum* 11.71%	*Lotus* – group 8.43%, Apiaceae A-type 6.56%, *Rubus* 5.62%	Unidentified 2 4.68%, *Urtica* 2.81%, Asteraceae S-form (*Carduus/Cirsium/Sarratula*) 2.34%, Brasicaceae 2.34%, *Tilia* 2.34%, Lamiaceae M-form (*Origanum, Thymus, Mentha, Melissa*) 2.11%, Poaceae 2.11%, Astaraceae J-form (*Centaurea jacea*) 1.87%, Ranunculaceae 1.64%, *Salix* 1.64%, Lamiaceae L-form (*Lamium*) 1.41%, *Echium* 1.17%, Liliaceae 1.17%, *Plantago* 1.17%	Apiaceae H-type 0.94%, Cistaceae 0.94%, *Trifolium pratense* -group 0.70%, *Rumex* 0.70%, *Clematis* 0.70%, Unidentified 11 0.70%, Others 0.05%
17	*Lotus*-group 52.14%	−	*Filipendula ulmaria* 11.92%	Fabaceae 7.26%	Rhamnaceae 3.72%, Apiaceae A/type 2.79%, Asteraceae J-form (*Centaurea jacea*) 2.23%, Polygonaceae 1.68%, Lamiaceae L-form (*Lamium*) 1.49%, Apiaceae H-type 1.30%, *Vicia* -type 1.30%, Liliaceae 1.30%, *Rubus* 1.30%, Lamiaceae M-form (*Origanum, Thymus, Mentha, Melissa*) 1.12%	Lamiaceae S-form (*Salvia*) 0.93%, *Teucrium* 0.93%, Ranunculaceae 0.93%, *Tilia* 0.74%, *Hypericum* 0.56%, Poaceae 0.56%, *Clematis* 0.56%, *Sanguisorba minor* 0.56%, Others 0.05%
18	−	*Filipendula ulmaria* 21.35%	Asteraceae J-form (*Centaurea jacea*) 12.81% *Rubus* 10.68%	*Trifolium repens* – group 7.83%, *Saxifraga* 5.69%, Ranunculaceae 5.34%	*Lotus* -group 3.56%, *Trifolium* 2.85%, *Hypericum* 2.85%, *Prunus/Malus/Pyrus* 2.85%, Apiaceae A-type 2.14%, Liliaceae 2.14%, Asteraceae – *Senecio* type (*Erigeron* form) 1.78%, Lamiaceae M – form (*Origanum, Thymus, Mentha, Melissa*) 1.78%, *Plantago* 1.78%, *Vicia* -type 1.42%, *Rumex* 1.42%, *Tilia* 1.42%, Apiaceae H – type 1.07%, Cyperaceae 1.07%, Unidentified 1 1.07%	*Robinia pseudoacacia* 0.71%, *Gleditshia* 0.71%, Lamiaceae S-form (*Salvia*) 0.71%, Others 0.03%
19	−	*Rubus* 25.07%	Asteraceae – *Senecio* type (*Erigeron* form) 14.67%	Asteraceae – *Senecio* type (*Senecio, Solidago*) 5.60%, *Lotus* – group 5.33%	Apiaceae A-type 4.00%, *Castanea sativa* 3.73%, Apiaceae H-type 3.47%, *Prunus/Malus/Pyrus* 3.20%, Fabaceae 2.67%, Ranunculaceae 2.67%, *Hypericum* 2.40%, Lamiaceae M-form (*Origanum, Thymus, Mentha, Melissa*) 2.40%, Lamiaceae L- form (*Salvia*) 1.33%, Asteraceae J-form (*Centaurea jacea*) 1.07%, Asteraceae T-form (*Taraxacum, Cichorium*) 1.07%, *Trifolium pratense* – group 1.07%, *Plantago* 1.07%, Unidentified 1.07%	*Artemisia* 0.80%, *Echium* 0.80%, Brasicacese 0.80%, *Trifolium repens* – group 0.80%, *Rumex* 0.80%, Unknown 6 0.80%, Unidentified 7 0.80%, Liliaceae 0.53%, *Sanguisorba minor* 0.53%, *Salix* 0.53%, *Saxifraga* 0.53%, *Urtica* 0.53%, Unidentified 2 0.53%, Others 0.03%
20	*Rubus* 45.42%	−	−	Apiaceae A-type 6.25	*Filipendula ulmaria* 4.79%, *Prunus/Malus/Pyrus* 4.58%, *Robinia pseudoacacia* 3.96%, Cistaceae 3.75%, *Plantago* 3.75%, Tetracolpate 2.92%, Fabaceae 2.50%, *Cornus sanguinea* 2.29%, Poaceae 2.29%, Ericaceae 1.46%, *Urtica* 1.46%, Apiaceae H – type 1.25%, *Salix* 1.25%, Rhamnaceae 1.04%	Asteraceae J – form (*Centaurea jacea*) 0.83%, Lamiaceae L – form (*Lamium*) 0.83%, *Clematis* 0.83%, Ranunculaceae 0.83%, *Saxifraga* 0.83%, *Lotus* – group 0.63%, Unidentified 8 0.63%, Others 0.05%
21	*Rubus* 49.71%	*Prunus/Malus/Pyrus* 21.22%	*Salix* 16.63%	*Robinia pseudoacacia* 5.93%	−	*Fraxinus ornus* 0.76%, Geum 0.76%, Cupressaceae 0.57%, Others 0.04%
22	−	*Rubus* 22.00%	−	Poaceae 6.00%, *Tilia* 5.78%, *Filipendula ulmaria* 5.11%	Unidentified 9 4.44%, Apiaceae H – type 3.56%, *Hypericum* 3.56%, Lamiaceae L – form (*Lamium*) 3.56%, *Urtica* 3.56%, Asteraceae – *Senecio* type (*Erigeron* form) 3.33%, *Trifolium repens* – group 3.33%, *Lotus* – group 2.44%, *Prunus/Malus/Pyrus* 2.44%, Unrecognizable 2.20%, Fabaceae 2.00%, *Clematis* 2.00%, Ranunculaceae 2.00%, Asteraceae – *Senecio* type (*Senecio, Solidago*) 1.78%, Unidentified 2 1.78%, Apiaceae A-type 1.56%, Tetracolpate 1.56%, Rhamnaceae 1.33%, Unidentified 10 1.33%, *Plantago* 1.11%	Cistaceae 0.89%, *Robinia pseudoacacia* 0.67%, Pinaceae 0.89%, *Potentilla/Fragaria* 0.89%, *Saxifraga* 0.89%, Asteraceae J – form (*Centaurea jacea*) 0.67%, Asteraceae – *Senecio* type (*Ambrosia* form) 0.67%, *Sanguisorba minor* 0.67%, Unidentified 8 0.67%, Others 0.05%
23	Ericaceae 70.11%	−	−	*Lotus* – group 6.93%	*Salix* 4.43%, *Hypericum* 2.95%, Fabaceae 2.16%, *Trifolium repens* – group 1.59%, *Prunus/Malus/Pyrus* 1.59%, Asteraceae S-form (*Carduus/Cirsium/Serratula*) 1.48%	Apiaceae A-type 0.91%, *Echium* 0.91%, *Rubus* 0.91%, *Potentilla/Fragaria* 0.91%, Asteraceae T-form (*Taraxacum, Cichorium*) 0.80%, Asteraceae – *Senecio* type (*Senecio, Solidago*) 0.57%, Others 0.04%
24	−	*Filipendula ulmaria* 27.08%	−	Fabaceae 8.89%, Ericaceae 8.04%, Apiaceae A – type 6.63%, Asteraceae – *Senecio* type (*Erigeron* form) 5.78%, *Rubus* 5.78%	*Plantago* 3.39%, *Prunus/Malus/Pyrus* 3.24%, Ranunculaceae 2.68%, Poaceae 2.26%, Tetracolpate 2.26%, *Lotus*-group 2.12%, *Hypericum* 2.12%, Asteraceae J – form (*Centaurea jacea*) 1.97%, Asteraceae T – form (*Taraxacum, Cichorium*) 1.55%, *Sanguisorba minor* 1.55%, *Robinia pseudoacacia* 1.41%, Lamiaceae L – form (*Lamium*) 1.41%, *Trifolium repens* – group 1.27%, Apiaceae H – type 1.13%	Lamiaceae M – form (*Origanum, Thymus, Mentha, Melissa*) 0.99%, *Salix* 0.99%, *Trifolium pratense* – group 0.56%, Lamiaceae S – form (*Salvia*) 0.56%, *Rumex* 0.56%, Others 0.06%
25	−	−	Apiaceae A – type 12.65%, *Rubus* 10.47%	*Filipendula ulmaria* 8.70%, Asteraceae J – form (*Centaurea jacea*) 6.32%, *Hypericum* 5.34%, *Trifolium pratense* – group 5.14%	*Trifolium repens* – group 4.74%, Ranunculaceae 3.95%, *Lotus* – group 3.75%, Cistaceae 3.16%, Lamiaceae S-form (*Salvia*) 3.16%, *Plantago* 3.16%, Poaceae 2.96%, Liliaceae 2.57%, *Thalicrum* 2.37%, Asteraceae S-form (*Carduus/Cirsium/Serratula*) 2.17%, *Prunus/Malus/Pyrus* 2.17%, Lamiaceae L – form (*Lamium*) 1.98%, *Clematis* 1.38%, *Tilia* 1.19%, *Urtica* 1.19%, Unidentified 1.19%	*Teucrium* 0.99%, *Salix* 0.99%, Unidentified 2 0.99%, Asteraceae – *Senecio* type (*Senecio, Solidago*) 0.79%, Ericaceae 0.79%, Fabaceae 0.59%, Lamiaceae M – form (*Origanum, Thymus, Mentha, Melissa*) 0.59%, *Potentilla/Fragaria* 0.59%, Others 0.04%
26	−	*Filipendula ulmaria* 22.92%	*Rubus* 11.91%, Fabaceae 10.11%	Apiaceae A-type 6.86%, Asteraceae J – form (*Centaurea jacea*) 5.60%	*Tilia* 3.79%, *Lotus* – group 3.25%, *Plantago* 2.89%, Apiaceae H – type 2.35%, *Thalicrum* 2.35%, *Ranunculaceae* 2.35%, *Poaceae* 2.17%, *Rumex* 2.17% *Prunus/Malus/Pyrus* 1.99%, *Hypericum* 1.81%, *Urtica* 1.62%, Asteraceae T-form (*Taraxacum, Cichorium*) 1.44%, *Trifolium pratense* – group 1.44%, Lamiaceae S- form (*Salvia*) 1.44%, Lamiaceae M- form (*Origanum, Thymus, Mentha, Melissa*) 1.08%	*Trifolium repens* – group 0.90%, Liliaceae 0.90%, Rhamnaceae 0.90%, *Saxifraga* 0.90%, Ericaceae 0.72%, *Robinia pseudoacacia* 0.72%, Asteraceae H – form (*Helianthus*) 0.54%, Lamiaceae L – form (*Lamium*) 0.54%, Unidentified 0.54%, Others 0.04%

Seven samples with a content of the particular pollen type exceeding 45% (Nos. 2, 10 13, 17, 20, 21, and 23) could generally be classified as monofloral honey (2). However, to confirm their classification other parameters such as electrical conductivity needed to be considered (1). Based on electrical conductivity (section 3.3. Physicochemical Parameters) two (Nos. 20 and 23) of those seven samples and three additional samples (Nos. 16, 22, and 25) were classified as honeydew honey (Table 3). Therefore, four samples were classified as monofloral (Nos. 2, 13, 17, and 21) and the remaining majority of the samples as polyfloral honey (Nos. 1, 3, 4, 5, 6, 7, 8, 9, 10, 11, 12, 14, 15, 18, 19, 24, and 26).

In the five monofloral samples the following pollen types exceeded 45%: Ericaceae family (No. 2), *Rubus* pollen (Nos. 13 and 21) and *Lotus*-group (No. 17) (Table 4). Five honeydew honey samples had high content of pollen particles of Ericaceae family (more than 70% in No. 23), as well as *Rubus* (more than: 45% in No. 20; 22% in No. 22, 10% in No. 25), Fabaceae (more than 15% in No. 16), *Filipendula ulmaria* (more than 13% in No. 16), Apiaceae A-type (more than 12% in No. 25) and *Hypericum* (more than 11% in No. 16) (Table 4). This was expected considering plant species that are mainly present in forests in the Tara Mountain region where bees collected honeydew such as blueberries (Ericaceae family), or blackberries and raspberries (*Rubus*) or weeds (*Hypericum*).

As can be seen in Table 4 polyfloral honey samples contained pollen particles mainly from *Rubus* (more than: 10% in Nos. 3, 4, 5, 6, 8, 9, 18, 26; 20% in Nos. 11, 12, 14, 19; 30% in No. 1), Ericaceae (more than: 30% in No. 5), *Filipendula ulmaria*: (more than: 10% in Nos. 1, 8, 9, 15; 20% in Nos. 11, 18, 24, 26, 50% in No. 10), Fabaceae (more than: 10% in Nos. 14, 26; 20% in Nos. 6, 3), *Hypericum* (more than: 10% in No. 12; 20% in No. 7), *Clematis* (more than: 10% in No. 6; 30 in No. 9), Asteraceae J – form (*Centaurea jacea*) (more than 10% in Nos. 8, 18), Asteraceae *Senecio* type (*Erigeron* form) (more than 10% in No. 19), *Plantago* (more than 10% in No. 4), *Robinia pseudoacacia* (more than 10% in No. 4), *Lotus* – group (more than 10% in No. 15) and *Salix* (more than 10% in No. 14). These results are consistent with the earlier report (6) that the species of the Fabaceae and Lamiaceae families contribute the greatest to the bee pasture in the Tara Mountain region.

Based on the results of the melissopalynological analyses, it can be noticed that most of the honey samples belong to polyfloral honey. Detailed pollen analyses showed a significant proportion of different pollen particles in all samples. This was expected for polyfloral honey samples, but it was also noted in monofloral and honeydew honey samples. Relying on diversity of pollen content in different types of honey (polyfloral, monofloral, and honeydew honey) the contribution of the diverse plant flora found on Tara Mountain could be seen.

### Physicochemical Parameters

Physicochemical parameters (pH, diastase activity, electrical conductivity, acidity, 5-(hydroxymethyl)furfural (5-HMF), glucose and fructose content, moisture content and water insoluble impurities content) of the 27 samples are summarized in Table 3. Applying the European Council’s criterion of electrical conductivity higher than 0.8 mS/cm for the honeydew honey (1), five samples (Nos. 16, 20, 22, 23, and 25) were classified as honeydew honey. The same classification of the honeydew honey was also used by other authors (8, 16–19). The 22 remaining honey samples were subsequently classified as blossom honey. Their ranges of electrical conductivity were 0.36–0.61 mS/cm for monofloral honey and 0.27–0.75 mS/cm for polyfloral honey (Table 3). Similar electrical conductivity ranges for polyfloral honey were published by other authors (8, 28).

In addition to having higher electrical conductivity, honeydew honey samples also had higher pH values than the blossom honey samples. Values for other parameters were higher for blossom honey samples than for honeydew honey samples (Table 3). Values of all determined parameters for the 27 honey samples were in accordance with international requirements (1): diastase activity ≥8 Schade units; acidity ≤50 meq/kg; 5-HMF ≤ 40 mg/kg; moisture content <20%; water insoluble impurities ≤0.1 g/100 g; sum of the glucose and fructose contents ≥60 g/100 g for blossom honey and ≥45 g/100 g for honeydew honey.

The highest average acidity content (Table 3) was measured for polyfloral honey samples (22.89 meq/kg) and the lowest for monofloral honey samples (19.93 meq/kg). Similar patterns were also observed in sensory analyses for sourness as a flavor attribute where several polyfloral honey samples were labeled as very/extremely/intensely sour (Table 2). The acidity content gives evidence of the presence of amino acids as well as the sugar fermentation process (29).

The highest average sum of glucose and fructose content was determined for monofloral honey (72.59 g/100 g) and the lowest for honeydew honey samples (66.10 g/100 g) (Table 3). The same pattern was also observed for the average content of sucrose (Table 3). There were no significant differences between the sugar contents in different types of honey (Table 3). The obtained contents of glucose and fructose in the honey samples were similar to the results for blossom and honeydew honey in another study (8).

For polyfloral honey samples the contents of 5-HMF ranged from 5.00 to 14.90 mg/kg (Nos. 3, 6, 7, 11, 12, 14, 18, and 19), while for monofloral honey the contents were 6.50 and 16.90 mg/kg (Nos. 13 and 21). The contents of 5-HMF were below the detection limit in the rest of monofloral and polyfloral, as well as all honeydew samples (Table 3). Another study reported higher contents of 5-HMF in polyfloral honey than in honeydew honey from Montenegro (8).

The results of physicochemical parameters of analyzed samples were in agreement with the literature data (8, 28) for different types of honey.

### Antioxidant Capacity of Honey Samples

**FIGURE 2:**
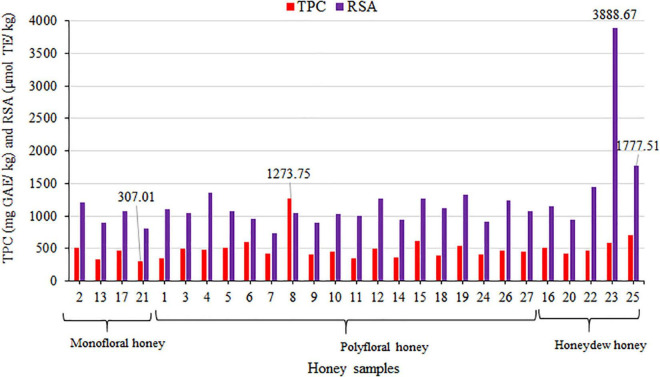
Distribution of the TPC (mg GAE/kg) and RSA (μmol TE/kg) in the analyzed honey samples from the Tara Mountain region in Serbia.

Antioxidant activities expressed through the TPCs and RSA were presented in Table 5. The TPC and RSA values obtained were grouped into the monofloral, polyfloral and honeydew honey types, as shown in Figure 2, and their descriptive analyses could be seen in Supplementary Table 1. Results of the antioxidant capacity of the samples showed similarity among most of the samples. The highest TPC was found in sample No. 8 (1273.75 ± 6.25 mg GAE/kg) and the highest RSA in sample No. 23 (3888.67 ± 53.99 μmol TE/kg), followed by No. 25 (1777.51 ± 20.58 μmol TE/kg) (Figure 2). The average value of RSA for honeydew honey was almost two times higher than for floral samples. The average values of the TPC and RSA were the highest for honeydew honey and the lowest for monofloral honey (Supplementary Table 1). The TPC for sample No. 8 was the only exception. These TPC and RSA values were similar to the results of other studies (8, 10, 30), with the additional observation of greater variability of the TPC and RSA values for polyfloral samples (8). Due to the similar color of most honey samples (mainly amber, Table 2), results of antioxidant activity were not entirely correlated with the honey color as suggested by other authors (11). Nevertheless, the obtained range of TPC (from 307.01 ± 4.77 to 1273.75 ± 6.25 mg GAE/kg) was comparable with the results obtained for buckwheat honey (26), which is recognizable by its specific dark color. These values were lower than the published TPC values for polyfloral and monofloral Algerian honey (14).

**TABLE 5:** Content of the 19 phenolic compounds (mg/kg), sum of the content of all 19 phenolic compounds (SUM-19; mg/kg), TPC (mg GAE/kg), and RSA (μmol TE/kg) in the analyzed honey samples (monofloral Nos. 2, 13, 17, and 21; polyfloral and honeydew honey Nos. 16, 20, 22, 23, and 25) from the Tara Mountain region in Serbia.

No.	PA	SA	ChA	CA	A	R	*p*C	Q3*O*G	EA	Q3*O*R	E	L	Q	N	K	H	I	P	G	SUM-19	TPC	RSA
1	1.85	1.24	0.35	3.93	2.71	0.26	10.12	0.03	2.51	0.13	0.13	0.19	1.72	0.24	1.78	0.43	3.51	5.86	2.47	39.46	353.64	1111.98
2	0.30	0.16	0.52	1.89	1.26	0.29	23.23	0.04	0.44	0.59	0.21	0.23	5.12	0.38	1.13	0.18	1.63	0.58	0.41	38.59	517.99	1212.92
3	0.50	0.52	0.43	3.91	2.83	0.54	21.01	0.07	2.65	0.14	0.31	0.42	1.63	0.37	2.45	0.62	3.27	3.37	1.01	46.05	498.57	1043.29
4	0.51	0.37	0.29	2.94	2.07	0.52	15.87	0.06	4.50	0.18	0.35	0.28	1.66	0.63	1.48	0.24	3.32	4.37	1.64	41.28	477.10	1350.09
5	1.00	1.73	0.35	6.83	4.82	0.38	17.55	0.05	0.70	0.16	0.40	0.37	3.82	1.54	3.27	0.87	3.93	7.88	3.92	59.57	506.10	1071.03
6	0.33	0.69	0.44	2.97	1.79	0.57	39.01	0.10	2.40	1.36	0.30	0.42	2.42	0.42	2.08	0.44	0.97	2.03	0.73	59.47	604.58	957.60
7	0.78	1.44	0.78	3.76	2.12	0.26	38.47	0.03	0.87	0.13	0.20	0.26	1.23	0.72	1.83	1.18	1.64	0.96	0.62	57.28	429.84	730.32
8	0.43	0.71	0.39	2.40	1.56	0.19	42.10	0.05	1.36	0.16	0.37	0.49	1.08	0.26	2.05	1.77	1.79	1.76	0.71	59.63	1273.75	1048.81
9	1.14	1.07	0.26	3.33	1.81	0.34	8.69	0.05	0.57	0.17	0.10	0.07	1.09	0.18	1.49	0.43	2.11	1.71	0.48	25.09	410.18	904.19
10	1.93	2.40	0.39	3.51	2.21	0.29	15.32	0.05	0.97	0.22	0.12	0.08	1.19	0.58	1.94	0.16	2.37	1.78	0.66	36.17	449.89	1027.78
11	0.69	0.86	0.39	2.96	1.61	0.53	23.42	0.05	1.05	0.14	0.12	0.09	1.15	0.63	1.68	0.33	2.03	1.62	0.63	39.98	345.43	999.23
12	1.04	1.44	0.56	3.94	2.29	0.29	38.24	0.08	2.67	0.32	0.21	0.30	2.29	0.54	2.77	1.45	3.44	5.36	2.49	69.72	504.20	1272.77
13	1.68	1.63	0.44	2.47	1.45	0.30	12.61	0.03	0.99	0.16	0.10	0.12	1.04	0.32	1.44	0.20	2.28	1.98	0.68	29.92	341.63	904.90
14	0.85	0.88	0.32	5.59	3.57	0.29	15.49	0.07	1.59	0.19	0.16	0.25	1.65	0.43	2.36	0.51	2.75	6.45	2.50	45.90	365.43	938.42
15	2.44	3.05	1.42	3.63	1.94	0.20	114.41	0.06	0.50	0.25	0.20	0.41	2.04	0.57	2.72	1.31	2.25	1.90	0.85	140.15	610.26	1263.50
16	2.70	2.70	0.85	2.91	1.56	0.20	72.12	0.05	0.34	0.19	0.10	0.26	1.49	0.44	1.51	0.42	1.40	0.36	0.38	89.98	505.17	1153.40
17	2.38	1.67	0.20	2.28	1.14	0.04	29.91	0.05	0.09	0.17	0.16	0.18	0.48	0.28	0.90	0.83	1.41	3.05	0.97	46.19	470.76	1069.60
18	1.05	1.24	0.81	4.60	2.71	0.25	22.26	0.04	1.56	0.18	0.17	0.29	1.24	0.49	2.74	1.49	3.29	2.99	1.23	48.63	392.13	1120.17
19	2.98	3.38	0.33	3.10	1.91	0.14	8.45	0.03	0.45	0.17	0.08	0.11	0.78	0.36	1.28	0.18	2.34	3.21	1.06	30.34	546.92	1329.06
20	0.80	1.11	0.79	16.96	10.09	0.36	39.28	0.14	3.40	0.30	0.45	0.65	4.75	0.79	6.85	1.65	3.54	12.07	7.30	111.28	416.67	942.55
21	0.19	0.24	0.87	5.02	2.85	0.39	14.60	0.03	0.34	0.12	0.10	0.17	1.21	0.48	2.53	0.50	2.51	7.16	2.42	41.73	307.01	812.06
22	2.25	2.58	0.55	9.80	5.57	0.21	25.89	0.06	1.79	0.22	0.26	0.30	4.04	1.51	2.95	0.71	5.07	8.17	2.91	74.84	462.16	1451.02
23	1.46	0.97	0.88	8.02	5.13	0.20	39.07	0.04	0.36	0.23	0.20	0.38	4.31	0.59	3.23	0.94	3.70	11.16	4.68	85.55	583.30	3888.67
24	1.83	2.98	0.97	7.97	4.73	0.38	33.02	0.07	1.17	0.18	0.27	0.40	0.83	0.88	3.28	0.63	3.54	9.10	3.22	75.45	404.12	911.15
25	2.42	3.02	0.53	3.57	1.82	0.20	27.30	0.05	9.86	0.20	0.21	0.28	2.21	0.43	2.18	0.82	2.67	3.07	1.26	62.10	703.13	1777.51
26	1.71	1.52	0.62	4.42	2.05	0.25	24.76	0.04	1.02	0.20	0.14	0.22	1.42	0.36	1.88	0.55	2.63	1.06	0.61	45.46	466.55	1242.73
27	1.06	1.55	0.56	4.61	2.78	0.45	30.14	0.04	0.99	0.13	0.32	0.41	1.58	0.29	1.96	0.41	3.27	4.06	1.29	55.90	452.29	1070.62

*19 phenolic compounds: aesculetin (A), caffeic acid (CA), chlorogenic acid (ChA), ellagic acid (EA), eriodictyol (E), galangin (G), hispidulin (H), isorhamnetin (I), kaempferol (K), luteolin (L), naringenin (N), p-coumaric acid (pC), pinocembrin (P), protocatechuic acid (PA), quercetin (Q), quercetin 3-O-glucoside (Q3OG), quercetin 3-O-rhamnoside (Q3OR), rutin (R), syringic acid (SA).*

### Polyphenols Quantification

In the honey samples investigated, 19 phenolic compounds were quantified using UHPLC-DAD MS/MS (Table 5). Among the 19 phenolic compounds, six were phenolic acids, while other compounds belonged to the group of flavonoids and their glycosides.

In monofloral, polyfloral and honeydew honey samples the highest contents were determined for *p-*coumaric acid (Table 5). In most of the samples *p-*coumaric acid and caffeic acid contributed the highest share of the sum of the content of all 19 phenolic compounds (SUM-19) (Table 5). The only exceptions were samples Nos. 2 and 4 in which *p-*coumaric acid and quercetin, as well as *p-*coumaric acid and isorhamnetin, respectively, had the highest share of the SUM-19 (Table 5). In most samples (15 of 27) the third highest share of content belonged to pinocembrin (Table 5), known as propolis-derived polyphenol (31). In the remaining 12 samples the third highest contents belonged to quercetin (Nos. 2 and 6), aesculetin (No. 7), kaempferol (Nos. 8 and 15), isorhamnetin (Nos. 9, 10, 11, 13, 18, and 26) and protocatechuic acid (No. 16). In contrast, chlorogenic acid, syringic acid, eriodictyol, luteolin, rutin, naringenin, hispidulin, quercetin 3-*O*-rhamnoside and quercetin 3-*O*-glucoside were found in lower concentrations. In most samples aesculetin had the fourth highest share and kaempferol the fifth. Among all samples, the highest average contents of caffeic acid, pinocembrin, aesculetin were detected in honeydew honey. Honeydew honey sample No. 20 contained the highest concentrations of caffeic acid, aesculetin, kaempferol, pinocembrin, and galangin (Table 5). The highest content of ellagic acid was found in honeydew honey sample No. 25 and the highest content of *p*-coumaric acid was found in honeydew honey sample No. 15 (114.41 mg/kg) (Table 5).

Among the three honey classes, the highest average content of phenolic compounds (SUM-19) (Table 5) was found in honeydew honey, which also had the highest average TPC and RSA (Supplementary Table 1). The highest SUM-19 was found in polyfloral honey sample No. 15, which was followed by three honeydew honey samples (Nos. 20, 16, and 23) (Table 5).

A comparison of monofloral, polyfloral and honeydew honey showed clear correlations between the SUM-19 (Supplementary Table 1), TPC and RSA. The correlations become less evident within each group of honey, even though the correlation remains the same for the first 10 samples containing the highest values. In all cases most of the honeydew honey samples are among the 10 samples with the highest SUM-19 (Nos. 20, 16, 23, 22, 25 – from highest to lowest SUM-19), TPC (Nos. 25, 23, 16 – from highest to lowest TPC values) and RSA (Nos. 23, 25, 22 – from highest to lowest RSA values) (Table 5). The correlation became less obvious, when comparing samples of the same honey type, showing the diversity of the samples within each of the groups (monofloral, polyfloral and honeydew honey).

The average sums of the contents of the 19 phenolic compounds were the lowest for monofloral honey samples (Supplementary Table 1). However, in monofloral honey sample No. 2, in which the prevailing pollen particles originated from the Ericaceae family (59.55%, Table 4), the content of quercetin (5.12 mg/kg, Table 5) was the highest among all 27 samples analyzed.

Similarly, honeydew honey sample No. 23, with a high content of pollen particles of Ericaceae family (Table 4) also had a high content of quercetin (4.31 mg/kg, Table 5), as well as pinocembrin and galangin (Table 5), which are, respectively, pollen-derived and propolis-derived flavonoids (31). Honeydew honey sample No. 23 had the highest RSA and high TPC values (Figure 2 and Table 5). Contents of several of the 19 phenolic compounds analyzed (Table 5) were within the same order of magnitude as reported for different types of honey by other authors (8, 10, 12, 32, 33).

The average content of phenolic compounds as well as the average values of TPC and RSA were the highest for honeydew honey and the lowest for monofloral honey (Supplementary Table 1). Comparing the contents of phenolic compounds with the antioxidant activities (Table 5 and Supplementary Table 1), it was noted that there was no considerable interdependence. However, many other phenolic compounds which were not observed might be found in these samples and also contribute to the phenolic profiles of honey and its antioxidant activity, which was also noted by other authors (8, 10, 12, 13, 34).

### Antimicrobial Activity of Honey Samples

Bacteriostatic action on *Escherichia coli* was observed for most of the 27 samples (Supplementary Table 2). A decrease in the number of microorganisms was found around the well (reservoir) in which the honey sample was located, but *Escherichia coli* was not eliminated. The majority of honey samples (16 of 27) were bactericidal for *Staphylococcus aureus* (Supplementary Table 2). There was no inhibition zone for *Candida albicans* (Supplementary Table 2). All honeydew honey samples (Nos. 16, 20, 22, 23, and 25) showed inhibition zones for *Staphylococcus aureus* (Supplementary Table 2). Honeydew honey sample No. 25 showed the largest inhibition zone. The results for monofloral and polyfloral honey samples were diverse: half showed inhibitory activity for *Staphylococcus aureus*. The average size of inhibition zones was largest for honeydew honey compared to monofloral and polyfloral honey. This might have been expected, as honeydew honey is made from honeydew which originates from aphids and trees. Other authors have reported enhanced antimicrobial activity of honeydew honey (35). In this study all samples had comparable sugar contents (glucose, fructose, sucrose – Table 3). Combining this data with the information on antimicrobial activity (Supplementary Table 2) showed that sugar content was not a deciding factor for antimicrobial activity. This was evident in the case of sample No. 21 which had the highest sugar content, but showed no inhibition zones. Furthermore, the average sugar content in honeydew honey samples was even slightly lower than in monofloral and polyfloral honey samples where sugar contents are comparable and the antimicrobial activity was only present in half of the samples.

Reviews of the literature record diverse observations of the antimicrobial activity of honey. Effects against the *Candida* and *Staphylococcu*s species were reported in a study of microbial diversity of fermentation in buckwheat honey (36). *Eucalyptus* honey showed no inhibition of *Candida* growth (37). Honey was proven effective against *Staphylococcus pyogenes* and *Escherichia coli* (37). Sensitivity of *Staphylococcus aureus* bacteria to different types of monofloral honey was reported (37). Observations for *Staphylococcu*s *aureus* in this study (Supplementary Table 2) were in agreement with published results of antibacterial activity of different honey types (33). Specific physicochemical parameters (such as low pH), the content of polyphenols and antioxidant activity contribute to the antimicrobial activity of honey (37). The inhibition of the bacteria *Staphylococcus aureus* (inhibition zones) and *Escherichia coli* (decreasing number of microorganisms) emphasizes honey’s potential for human nutrition. Although data in the literature (36, 37) gives strong evidence for antimicrobial activity of honey against *Staphylococcu*s *aureus, Candida* and *Escherichia coli*, the 27 honey samples examined in this study showed bactericidal activity only against *Staphylococcu*s *aureus* and to some extent a bacteriostatic activity against *Escherichia coli*. These antimicrobial activities combined with antioxidant activity show the potential of honey from the Tara Mountain region for human nutrition and health benefits.

### Chemometric Analyses

#### Chemometric Analyses of Melissopalynological Analyses Results

The results of the melissopalynological analyses provided a good differentiation of honey samples. Chemometric methods were then used to confirm the results obtained and also to present and interpret the results in a different way. The attempt was to classify honey samples to monofloral, polyfloral and honeydew using chemometric methods. The phylogenetic tree diagram was created using R software 4.0.2 (64-bit version). The R package “ape” (Analysis of Phylogenetics and Evolution) was applied as a graphical tool to represent melissopalynology analyses data, assessed in the cluster analysis. The experimental results obtained were presented in the resulting matrix, after which the hierarchical cluster analysis was performed. The distance matrix was determined using the Euclidean method, while the cluster analysis was performed using the “complete” method.

**FIGURE 3:**
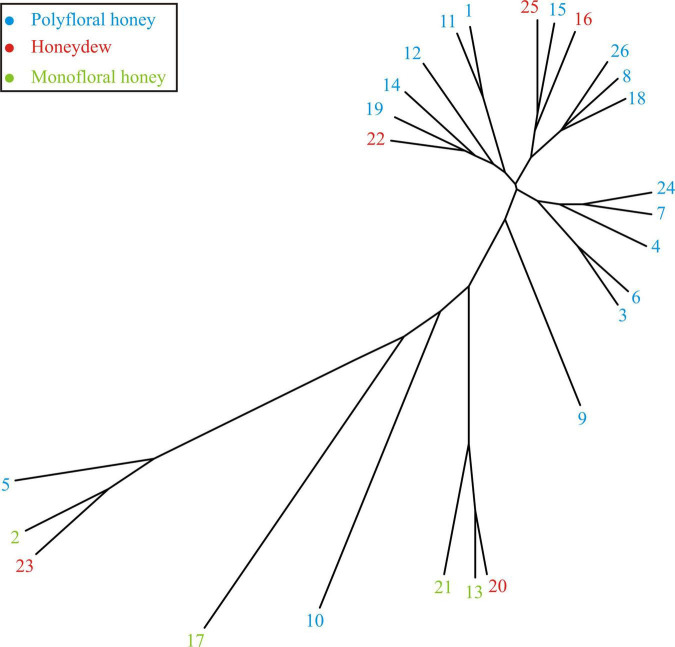
The unrooted phylogenetic tree based on the melissopalynological analyses results presented in Table 4.

The similarities of the honey samples could be observed according to the vicinity of branches presented in Figure 3. Several clusters were observed. The first cluster was formed by samples Nos. 1, 11, 12, 14, 19, and 22, with *Rubus* pollen content higher than 22% and lower than 32% (Table 4). The second cluster was formed by samples with lower *Rubus* pollen content, but also with *Filipendula ulmaria* pollen content higher than 8.70% and lower than 23% (Table 4). The third cluster was formed by samples Nos. 3, 4, 6, 7, and 24 with different pollen particles, such as pollen of Fabaceae family, *Hypericium* or *Filipendula ulmaria* (Table 4) content ranging from 20–30%. The fourth cluster was formed by samples Nos. 2, 5, and 23, with obvious dominant content of pollen particles from Ericaceae family (from 38.21 to 70.11%) (Table 4). The fifth cluster was formed by samples Nos. 13, 20 and 21 with *Rubus* pollen content higher than 45% (Table 4). The remaining samples (Nos. 9, 10, and 17) did not belong to any of these clusters due to their different content of pollen particles (Table 4).

Despite the obtained clusters for most of the honey samples the separation between monofloral (Nos. 2, 13, 17, and 21) and honeydew honey (Nos. 16, 22, and 25) could be observed. The exceptions were honeydew honey samples Nos. 20 and 23, which were more similar to the monofloral honey type due to their high content of pollen from one plant species (Table 4). Polyfloral honey samples (Nos. 1, 3, 4, 5, 6, 7, 8, 9, 10, 11, 12, 14, 15, 18, 19, 24, and 26) were expected to be between these two groups.

#### Chemometric Analyses of Physicochemical Results

**FIGURE 4:**
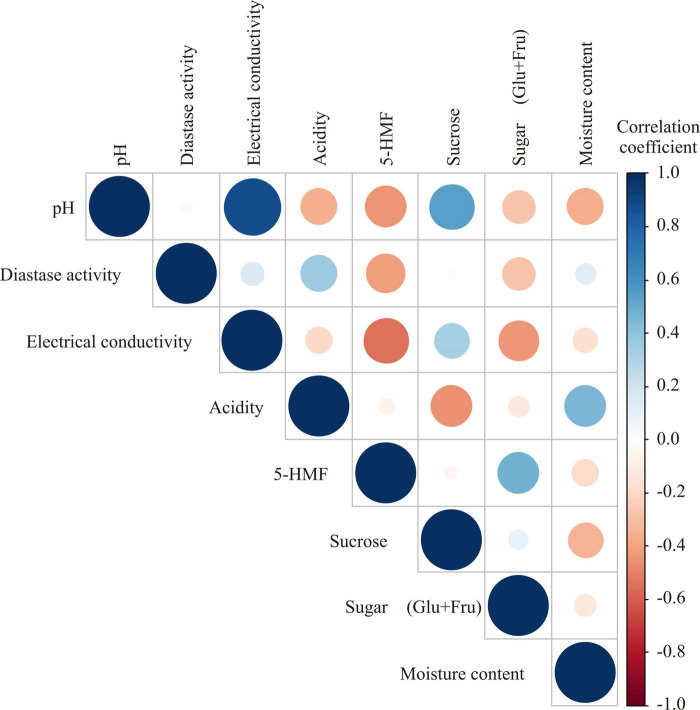
Color correlation graph between physicochemical parameters in honey samples.

In order to obtain better differentiation of honey, chemometric analyses were additionally performed on physicochemical parameters. The correlations are presented by color graph (Figure 4). Statistically significant correlations (*p* ≤ 0.01) were found between several physicochemical parameters (Figure 4). The circle’s color is defined by the correlation coefficient value, while the circle’s size is defined by the *p* – value of the correlation. The highest positive correlations were found between pH values and electrical conductivity (*r* = 0.882, *p* ≤ 0.001). This can be explained by the fact that electrical conductivity of honey is highly dependent on the content of minerals, salts, as well as organic acids, which affect the pH value. Positive correlations were also noted between pH and sucrose content (*r* = 0.542, *p* ≤ 0.01), while electrical conductivity was negatively correlated to 5-HMF content (*r* = −0.543, *p* ≤ 0.01).

The PCA of the physicochemical parameters of honey samples (Supplementary Figure 1) explained that the first three principal components account for 74.55% of the total variance in the 8 parameters (pH, diastase activity, electrical conductivity, acidity, 5-HMF, sucrose, sugar (Glu + Fru) and moisture content). According to the results of the PCA, the content of 5-HMF (which contributed 11.6% of the total variance, based on correlations), exhibited a positive influence on the first principal component (PC1), while pH value (30.4%), electrical conductivity (27.8%) and sucrose content (11.9%) negatively affected the calculation of PC1. Diastase activity (18.7% of the total variance, based on correlations), acidity (20.5%) and moisture content (16.3%) showed a positive influence on the second principal component (PC2), while 5-HMF (16.9%), sucrose (9.2%), and sugar content (16.2%) exerted a negative score according to the PC2 component. Moisture content (9.9% of the total variance, based on correlations) showed a positive influence on the third principal component (PC3) calculation, while diastase activity (57.5%), sucrose (16.4%), and sugar content (10.1%) exerted a negative influence on PC3 (Supplementary Figure 1). The results of applied chemometrics complemented the previously observed differences between honey samples (Table 3).

#### Chemometric Analyses of the Results of Phenolic Compounds Analyses, Antioxidant and Antimicrobial Activity

**FIGURE 5:**
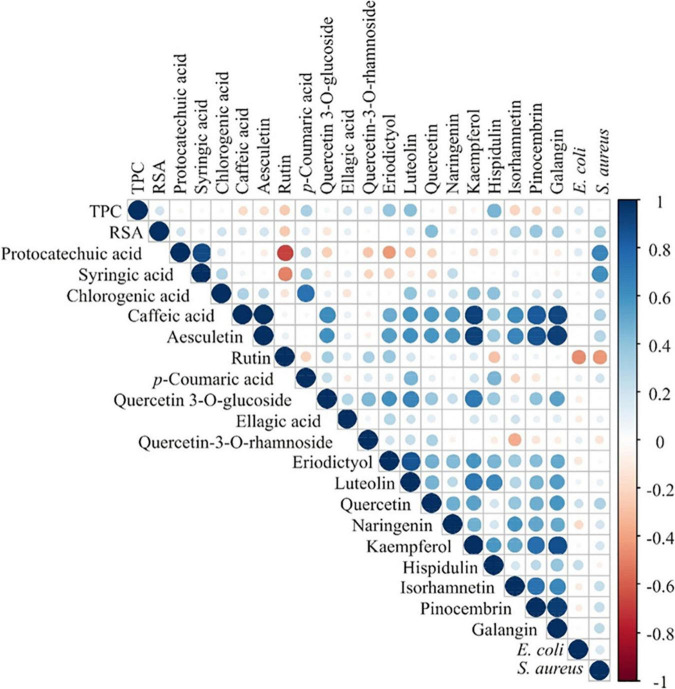
Color correlation graph between the content of phenolic compounds, antioxidant activity (TPC and RSA) and antimicrobial activity.

The results of complementary analyses of the contents of phenolic compounds, antioxidant capacity and antimicrobial activity have the potential to provide additional information on the botanical and biological origin of honey. Correlation analysis of these results is presented in Figure 5. The highest positive correlations at *p* ≤ 0.001 were between the contents of protocatechuic acid and syringic acid, caffeic acid and aesculetin (*r* = 0.989), chlorogenic acid and *p*-coumaric acid (*r* = 0.738). Luteolin content and the contents of quercetin 3-*O*-glucoside and eriodictyol were positively correlated (*r* = 0.650 and 0.853, respectively). Kaempferol content was positively correlated to the contents of caffeic acid, aesculetin, quercetin 3-*O*-glucoside and luteolin (*r* = 0.927; *r* = 0.926; *r* = 0.707; *r* = 0.713, respectively). Isorhamnetin content was positively correlated to aesculetin content (*r* = 0.652). Pinocembrin content was positively correlated to contents of caffeic acid, aesculetin, kaempferol, and isorhamnetin (*r* = 0.834; *r* = 0.876, *r* = 0.759, and *r* = 0.735, respectively). Galangin content was positively correlated to the contents of caffeic acid, aesculetin, kaempferol, isorhamnetin and pinocembrin (*r* = 0.909 4; *r* = 0.937, *r* = 0.875, *r* = 0.650, and *r* = 0.949 respectively). Additionally, protocatechuic acid content was positively correlated to the inhibition zone of *S. aureus* (*r* = 0.660; *p* ≤ 0.001) (Figure 5).

The PCA of the phenolic content, antioxidant (expressed as TPC and RSA) and antimicrobial activity in honey samples (Supplementary Figure 2) explained that the first three principal components summarized 61.23% of the total variance in the 23 parameters (TPC, RSA, protocatechuic acid, syringic acid, chlorogenic acid, caffeic acid, aesculetin, rutin, *p*-coumaric acid, quercetin 3-*O*-glucoside, ellagic acid, quercetin 3-*O*-rhamnoside, eriodictyol, luteolin, quercetin, naringenin, kaempferol, hispidulin, isorhamnetin, pinocembrin, galangin, *E. coli* and *S. aureus*). The content of caffeic acid (11.3% of the total variance, according to correlations), aesculetin (11.7%), luteolin (7.8%), kaempferol (11.6%), pinocembrin (9.4%), and galangin (11.1%) showed a negative influence on the PC1 coordinate. The content of protocatechuic acid, syringic acid and *S. aureus* activity (22.5, 18.6, and 14.2%, respectively) showed a positive influence on PC2 coordinate computation, while the content of rutin (17.5%) exerted a negative influence on PC2 coordinate. The content of isorhamnetin (8.7%) showed a positive influence on the PC3 coordinate, whereas TPC as well as the contents of *p*-coumaric acid, luteolin and hispidulin (18.2; 19.1; 9.5, and 11.2%, respectively) showed a negative influence on PC3 coordinate (Supplementary Figure 2). Sample No. 20 stands out from the rest in PCA (Supplementary Figure 2) and also in chemical analyses of phenolic compounds with the highest content of caffeic acid, aesculetin, quercetin, kaempferol, pinocembrin, and galangin (Table 5).

Based on literature data the obtained correlations could serve as additional parameters for determining the geographical origin of honey samples. Caffeic acid derivatives and chlorogenic acid in polyfloral honey (10) as well as eriodictyol and quercetin in monofloral honey (29, 34) were reported as potential markers for Serbian honey. In addition to other phenolic compounds, quercetin and naringenin were proposed as potential markers for the botanical origin of honeydew honey (12). These compounds were found in all investigated honey samples from the Tara Mountain region and were present in higher amounts in honeydew honey samples.

## Conclusion

This study presents a detailed characterization of the honey samples from the Tara Mountain region in Serbia. A comprehensive study of the melissopalynological analyses provides insight to the origin of the honey, as well as the diverse plants visited by the bees. Together with the electrical conductivity, the results of melissopalynological analyses helped to classify four samples as monofloral, five samples as honeydew honey and the remaining samples as polyfloral honey. Physicochemical parameters (pH, diastase activity, acidity, content of 5-HMF, sucrose and moisture content) of all samples were in accordance with International requirements. Nineteen phenolic compounds (phenolic acids, flavonoids and their glycosides) were quantified in all honey samples. The average content of phenolic compounds as well as the average values of TPC and radical scavenging activities were the highest for honeydew honey and the lowest for monofloral honey. Honey samples showed antimicrobial effects on *Escherichia coli* (bacteriostatic activity) and *Staphylococcus aureus* (bactericidal activity). Chemometric analyses were performed using the following results: melissopalynological analyses, physicochemical analyses, phenolic content analyses and antioxidant activity. PCA resulted in the grouping of honey samples in factor space, based on the differences in the physicochemical parameters and polyphenols contents and antioxidant capacity. The honeydew samples are clearly isolated forming a cluster, emphasizing the highest electrical conductivity and pH value, showing that PCA analysis represents a good tool for their separation based on examined physicochemical parameters. Furthermore, chemometric analyses performed on the polyphenols content and antioxidant capacity showed that these parameters are valuable to provide enough information to distinguish the botanical origin of honey samples.

## Data Availability Statement

The original contributions presented in this study are included in the article/Supplementary Material, further inquiries can be directed to the corresponding author.

## Author Contributions

ŽT and NN conceived and designed the analyses. NN contributed to sample distribution. MN, UG, KJ, RB, PR, and LP performed the analyses. IV, MN, ŽT, and NN wrote the manuscript. ŽT, IV, and MN conceived and designed the manuscript. All authors read and approved the final manuscript.

## Conflict of Interest

The authors declare that the research was conducted in the absence of any commercial or financial relationships that could be construed as a potential conflict of interest.

## Publisher’s Note

All claims expressed in this article are solely those of the authors and do not necessarily represent those of their affiliated organizations, or those of the publisher, the editors and the reviewers. Any product that may be evaluated in this article, or claim that may be made by its manufacturer, is not guaranteed or endorsed by the publisher.

## References

[1] European Parliament. Directive 2014/63/EU of the European parliament and of the council amending council directive 2001/110/EC relating to honey. *OJEC*. (2014) L164. Available online at: https://www.legislation.gov.uk/eudr/2001/110/contents (accessed April 20, 2022).

[2] Von Der Ohe W Oddo L Piana M Morlot M Martin P. Harmonized methods of melissopalynology. *Apidologie.* (2004) 35:S18–25. 10.1051/apido:2004050

[3] Republic of Serbia. *Official Gazette Republic of Serbia No.101/15. Rulebook on the Quality of Honey, Honey Products and Other Bee Products.* (2013). Available online at: http://extwprlegs1.fao.org/docs/pdf/srb130173.pdf (accessed April 20, 2022).

[4] Stevanović V. *The Red Data Book of Flora of Serbia 1 – Extinct and Critically Endangered Taxa.* Belgrade: Ministry of Environment of the Republic of Serbia (1999).

[5] Tomićević J Bjedov I Gudurić I Obratov-Petković D Shannon AM. Tara national park – resources, management and tourist perception. In: Sladonja B editor. *Protected Area Management.* London: IntechOpen (2012). p. 240. 10.5772/51197

[6] Jarić S Mačukanović-Jocić M Mitrović M Pavlović P. The melliferous potential of forest and meadow plant communities on Mount Tara (Serbia). *Environ Entomol.* (2013) 42:724–32. 10.1603/EN13031 23905735

[7] Hawkins J de Vere N Griffith A Ford CR Allainguillaume J Hegarty MJ Using DNA metabarcoding to identify the floral composition of honey: a new tool for investigating honey bee foraging preferences. *PLoS One.* (2015) 10:e0134735. 10.1371/journal.pone.0134735 26308362 PMC4550469

[8] Nešović M Gašić U Tosti T Trifković J Baošić R Blagojević S Physicochemical analysis and phenolic profile of polyfloral and honeydew honey from Montenegro. *RSC Adv.* (2020) 10:2462–71. 10.1039/C9RA08783D 35496084 PMC9048719

[9] Lazarević KB Andrić F Trifković J Tešić Ž Milojković-Opsenica D. Characterisation of Serbian unifloral honeys according to their physicochemical parameters. *Food Chem.* (2012) 132:2060–4. 10.1016/j.foodchem.2011.12.048

[10] Gašić U Kečkeš S Dabić D Trifković J Milojković-Opsenica D Natić M Phenolic profile and antioxidant activity of Serbian polyfloral honeys. *Food Chem.* (2014) 145:599–607. 10.1016/j.foodchem.2013.08.088 24128520

[11] Bertoncelj J Doberšek U Jamnik M Golob T. Evaluation of the phenolic content, antioxidant activity and colour of Slovenian honey. *Food Chem.* (2007) 105:822–8. 10.1016/j.foodchem.2007.01.060

[12] Vasić V Gašić U Stanković D Lušić D Vukić-Lušić D Milojković-Opsenica D Tešić Ž Trifković J. Towards better quality criteria of European honeydew honey: phenolic profile and antioxidant capacity. *Food Chem.* (2019) 274:629–41. 10.1016/j.foodchem.2018.09.045 30372988

[13] Gašić UM Natić MM Mišić DM Lušić DV Milojković-Opsenica DM Tešić ŽL Lušić D. Chemical markers for the authentication of unifloral *Salvia officinalis* L. honey. *J Food Compos Anal.* (2015) 44:128–38. 10.1016/j.jfca.2015.08.008

[14] Otmani A Amessis-Ouchemoukh N Birinci C Yahiaoui S Kolayli S Rodríguez-Flores MS Phenolic compounds and antioxidant and antibacterial activities of Algerian honeys. *Food Biosci.* (2021) 42:101070. 10.1016/j.fbio.2021.101070

[15] Gašić UM Milojković-Opsenica DM Tešić ŽL. Polyphenols as possible markers of botanical origin of honey. *J AOAC Int.* (2017) 100:852–61. 10.5740/jaoacint.17-0144 28527184

[16] Can Z Yildiz O Sahin H Turumtay EA Silici S Kolayli S. An investigation of Turkish honeys: their physico-chemical properties, antioxidant capacities and phenolic profiles. *Food Chem.* (2015) 180:133–41. 10.1016/j.foodchem.2015.02.024 25766810

[17] Soria AC González M de Lorenzo C Martínez-Castro I Sanz J. Estimation of the honeydew ratio in honey samples from their physicochemical data and from their volatile composition obtained by SPME and GC-MS. *J Sci Food Agric.* (2005) 85:817–24. 10.1002/jsfa.1890

[18] Popek S Halagarda M Kursa K. A new model to identify botanical origin of Polish honeys based on the physicochemical parameters and chemometric analysis. *LWT Food Sci Technol.* (2017) 77:482–7. 10.1016/j.lwt.2016.12.003

[19] Pita-Calvo C Vázquez M. Differences between honeydew and blossom honeys: a review. *Trends Food Sci Technol.* (2017) 59:79–87. 10.1016/j.tifs.2016.11.015

[20] Bogdanov S. *Harmonised Methods of the International honey commission. Bee Product Science.* International Honey Commission (2009). Available online: https://www.ihc-platform.net/ihcmethods2009.pdf (accessed April 20, 2022).

[21] Pasias IN Raptopoulou KG Makrigennis G Ntakoulas DD Lembessis D Dimakis V Finding the optimum treatment procedure to delay honey crystallization without reducing its quality. *Food Chem.* (2022) 381:132301. 10.1016/j.foodchem.2022.132301 35124485

[22] Lazarević KB Jovetić MS Tešić ŽL. Physicochemical parameters as a tool for the assessment of origin of honey. *J AOAC Int.* (2017) 100:840–51. 10.5740/jaoacint.17-0143 28527181

[23] Koulis GA Tsagkaris AS Aalizadeh R Dasenaki ME Panagopoulou EI Drivelos S Honey phenolic compound profiling and authenticity assessment using HRMS targeted and untargeted metabolomics. *Molecules.* (2021) 26:2769. 10.3390/molecules26092769 34066694 PMC8125859

[24] Bugeja DA Nešović M Šikoparija B Radišić P Tosti T Trifković J Melissopalynology analysis, determination of physicochemical parameters, sugars and phenolics in Maltese honey collected in different seasons. *J Serbian Chem Soc.* (2022). 10.2298/JSC211214033B

[25] Becerril-Sánchez AL Quintero-Salazar B Dublán-García O Escalona-Buendía HB. Phenolic compounds in honey and their relationship with antioxidant activity, botanical origin, and color. *Antioxidants.* (2021) 10:1700. 10.3390/antiox10111700 34829570 PMC8614671

[26] Nešović M Gašić U Tosti T Horvacki N Šikoparija B Nedić N Polyphenol profile of buckwheat honey, nectar and pollen. *R Soc Open Sci.* (2020) 7:201576. 10.1098/rsos.201576 33489289 PMC7813236

[27] Kirste K Günther V Nikolaus K. Comparison of pollen spectra collected by four different subspecies of the honey bee *Apis mellifera*. *Apidologie.* (2007) 38:341–53. 10.1051/apido:2007020

[28] do Nascimento KS Sattler JAG Macedo LFL González CVS de Melo ILP da Silva Araújo E Phenolic compounds, antioxidant capacity and physicochemical properties of Brazilian *Apis mellifera* honeys. *LWT Food Sci Technol.* (2018) 91:85–94. 10.1016/j.lwt.2018.01.016

[29] Habib HM Al Meqbali FT Kamal H Souka UD Ibrahim WH. Physicochemical and biochemical properties of honeys from arid regions. *Food Chem.* (2014) 153:35–43. 10.1016/j.foodchem.2013.12.048 24491697

[30] Ciucure CT Geanã EI. Phenolic compounds profile and biochemical properties of honeys in relationship to the honey floral sources. *Phytochem Anal.* (2019) 30:481–92. 10.1002/pca.2831 31025476

[31] Tomás-Barberán FA Ferreres F Carcia-Viguera C Tomás-Lorente F. Flavonoids in honey of different geographical origin. *Z Lebensm Unters Forsch.* (1993) 196:38–44. 10.1007/BF01192982

[32] Halouzka R Tarkowski P Zeljković Ćavar S. Characterisation of phenolics and other quality parameters of different types of honey. *Czech J Food Sci.* (2016) 34:244–53. 10.17221/321/2015-CJFS

[33] Salonen A Virjamo V Tammela P Fauch L Julkunen-Tiitto R. Screening bioactivity and bioactive constituents of Nordic unifloral honeys. *Food Chem.* (2017) 237:214–24. 10.1016/j.foodchem.2017.05.085 28763988

[34] Kečkeš S Gašić U Veličković TĆ Milojković-Opsenica D Natić M Tešić Ž. The determination of phenolic profiles of Serbian unifloral honeys using ultra-high-performance liquid chromatography/high resolution accurate mass spectrometry. *Food Chem.* (2013) 138:32–40. 10.1016/j.foodchem.2012.10.025 23265452

[35] Jílková V Cajthaml T Frouz J. Relative importance of honeydew and resin for the microbial activity in wood ant nest and forest floor substrate–a laboratory study. *Soil Biol Biochem.* (2018) 117:1–4. 10.1016/j.soilbio.2017.11.002

[36] Bednarek M Szwengiel A Flórez AB Czarnecki Z Mayo B. Effect of different starter cultures on chemical and microbial parameters of buckwheat honey fermentation. *Food Microbiol.* (2019) 82:294–302. 10.1016/j.fm.2019.03.006 31027786

[37] Valdés-Silverio LA Iturralde G García-Tenesaca M Paredes-Moreta J Narváez-Narváez DA Rojas-Carrillo M Physicochemical parameters, chemical composition, antioxidant capacity, microbial contamination and antimicrobial activity of *Eucalyptus* honey from the Andean region of Ecuador. *J Apic Res.* (2018) 57:382–94. 10.1016/j.foochem.2018.09.045

