# Supplementary material for: Polyphenolic and Chemical Profiles of Honey From the Tara Mountain in Serbia
Source: Front Nutr. 2022 Jun 24;9:941463. doi: 10.3389/fnut.2022.941463 (PMC9263698; doi:10.3389/fnut.2022.941463)
Supplement: Supplementary file 1 [file Data_Sheet_1.DOCX]

*Supplementary Material*

**1.1. Supplementary Figures Legend:**

**Supplementary Figure S1.** PCA ordination of physicochemical parameters in honey samples: **(A)** projection in PC1-PC2 plane; **(B)** projection in PC1-PC3 plane.

**Supplementary Figure S2.** PCA ordination of the content of phenolic compounds, antioxidant activity (TPC and RSA) and antimicrobial activity variables: **(A)** projection in PC1-PC2 plane; **(B)** projection in PC1-PC3 plane. Abbreviations for 19 phenolic compounds: aesculetin (A), caffeic acid (CA), chlorogenic acid (ChA), ellagic acid (EA), eriodictyol (E), galangin (G), hispidulin (H), isorhamnetin (I), kaempferol (K), luteolin (L), naringenin (N), *p*-coumaric acid (*p*C), pinocembrin (P), protocatechuic acid (PA), quercetin (Q), quercetin 3-*O*-glucoside (Q3*O*G), quercetin 3-*O*-rhamnoside (Q3*O*R), rutin (R), syringic acid (SA).

**1.2. Supplementary Tables Legend:**

**Supplementary Table S1.** Description analysis of the content of phenolic compounds (mg/kg), total phenolic content (TPC) and relative scavenging activity (RSA) in the analyzed honey samples (monofloral Nos. 2, 13, 17, 21; polyfloral; and honeydew honey Nos. 16, 20, 22, 23, 25) from the Tara Mountain region in Serbia

**Supplementary Table S2.** Antimicrobial effects of analyzed honey samples obtained with the agar diffusion method.

**Table S1.** Description analysis of the content of phenolic compounds (mg/kg), total phenolic content (TPC) and relative scavenging activity (RSA) in the analyzed honey samples (monofloral Nos. 2, 13, 17, 21; polyfloral; and honeydew honey Nos. 16, 20, 22, 23, 25) from the Tara Mountain region in Serbia.

| **Parametere** | **Monofloral honey (Nos. 2, 13, 17, 21)** | | | |  | **Polyfloral honey** | | | |  | **Honeydew honey (Nos. 16, 20, 22, 23, 25)** | | | |
| --- | --- | --- | --- | --- | --- | --- | --- | --- | --- | --- | --- | --- | --- | --- |
|  | **Max** | **Min** | **Average** | **SD** |  | **Max** | **Min** | **Average** | **SD** |  | **Max** | **Min** | **Average** | **SD** |
| **Protocatechuic acid** | 2.38 | 0.19 | 1.14 | 1.07 |  | 2.98 | 0.19 | 1.26 | 0.77 |  | 2.70 | 0.80 | 1.93 | 0.78 |
| **Syringic acid** | 1.67 | 0.16 | 0.93 | 0.84 |  | 3.38 | 0.24 | 1.46 | 0.87 |  | 3.02 | 0.97 | 2.08 | 0.97 |
| **Chlorogenic acid** | 0.87 | 0.20 | 0.51 | 0.28 |  | 1.42 | 0.20 | 0.53 | 0.29 |  | 0.88 | 0.53 | 0.72 | 0.17 |
| **Caffeic acid** | 5.02 | 1.89 | 2.92 | 1.42 |  | 7.97 | 2.28 | 4.01 | 1.43 |  | 16.96 | 2.91 | 8.25 | 5.67 |
| **Aesculetin** | 2.85 | 1.14 | 1.68 | 0.79 |  | 4.82 | 1.14 | 2.43 | 0.97 |  | 10.09 | 1.56 | 4.83 | 3.47 |
| **Rutin** | 0.39 | 0.04 | 0.25 | 0.15 |  | 0.57 | 0.04 | 0.33 | 0.14 |  | 0.36 | 0.20 | 0.23 | 0.07 |
| ***p-*coumaric acid** | 29.91 | 12.61 | 20.08 | 8.01 |  | 114.41 | 8.45 | 27.40 | 22.59 |  | 72.12 | 25.89 | 40.73 | 18.65 |
| **Quercetin 3-*O*-glucoside** | 0.05 | 0.03 | 0.04 | 0.01 |  | 0.10 | 0.03 | 0.05 | 0.02 |  | 0.14 | 0.04 | 0.07 | 0.04 |
| **Ellagic acid** | 0.99 | 0.09 | 0.46 | 0.38 |  | 4.50 | 0.09 | 1.38 | 1.05 |  | 9.86 | 0.34 | 3.15 | 3.96 |
| **Quercetin 3-*O*-rhamnoside** | 0.59 | 0.12 | 0.26 | 0.22 |  | 1.36 | 0.12 | 0.23 | 0.26 |  | 0.30 | 0.19 | 0.23 | 0.04 |
| **Eriodictyol** | 0.21 | 0.10 | 0.14 | 0.05 |  | 0.40 | 0.08 | 0.21 | 0.10 |  | 0.45 | 0.10 | 0.25 | 0.13 |
| **Luteolin** | 0.23 | 0.12 | 0.17 | 0.05 |  | 0.49 | 0.07 | 0.26 | 0.13 |  | 0.65 | 0.26 | 0.38 | 0.16 |
| **Quercetin** | 5.12 | 0.48 | 1.96 | 2.13 |  | 3.82 | 0.48 | 1.50 | 0.72 |  | 4.75 | 1.49 | 3.36 | 1.42 |
| **Naringenin** | 0.48 | 0.28 | 0.37 | 0.09 |  | 1.54 | 0.18 | 0.50 | 0.29 |  | 1.51 | 0.43 | 0.75 | 0.45 |
| **Kaempferol** | 2.53 | 0.90 | 1.50 | 0.72 |  | 3.28 | 0.90 | 2.09 | 0.64 |  | 6.85 | 1.51 | 3.34 | 2.07 |
| **Hispidulin** | 0.83 | 0.18 | 0.43 | 0.30 |  | 1.77 | 0.16 | 0.69 | 0.48 |  | 1.65 | 0.42 | 0.91 | 0.46 |
| **Isorhamnetin** | 2.51 | 1.41 | 1.96 | 0.53 |  | 3.93 | 0.97 | 2.60 | 0.80 |  | 5.07 | 1.40 | 3.28 | 1.36 |
| **Pinocembrin** | 7.16 | 0.58 | 3.20 | 2.83 |  | 9.10 | 0.96 | 3.70 | 2.39 |  | 12.07 | 0.36 | 6.96 | 5.10 |
| **Galangin** | 2.42 | 0.41 | 1.12 | 0.90 |  | 3.92 | 0.48 | 1.44 | 0.99 |  | 7.30 | 0.38 | 3.30 | 2.77 |
| **Sum of phenolic compounds (mg/kg)** | 46.18 | 29.92 | 39.10 | 6.87 |  | 140.13 | 25.09 | 52.06 | 23.94 |  | 111.28 | 62.12 | 84.75 | 18.31 |
| **TPC (mg GAE/ kg)** | 517.99 | 307.01 | 409.35 | 101.05 |  | 1273.75 | 345.43 | 505.06 | 206.83 |  | 703.13 | 416.67 | 534.09 | 112.68 |
| **RSA (µmol TE/kg)** | 1212.92 | 812.06 | 999.87 | 177.52 |  | 1350.09 | 730.32 | 1077.37 | 165.08 |  | 3888.67 | 942.55 | 1842.63 | 1186.28 |

**Table S2.** Antimicrobial effects of analyzed honey samples obtained with the agar diffusion method.

| **Sample No.** | **Microorganisms** | | |
| --- | --- | --- | --- |
|  | ***Escherichia coli*** | ***Staphylococcus aureus*** | ***Candida albicans*** |
|  | **Inhibition zone (mm)** | | |
| **1** | /* | 15 | / |
| **2** | /* | 15 | / |
| **3** | /* | / | / |
| **4** | / | / | / |
| **5** | /* | 17 | / |
| **6** | /* | / | / |
| **7** | /* | / | / |
| **8** | /* | / | / |
| **9** | /* | 15 | / |
| **10** | /* | 17 | / |
| **11** | / | / | / |
| **12** | /* | / | / |
| **13** | /* | / | / |
| **14** | /* | / | / |
| **15** | /* | 20 | / |
| **16** | /* | 16 | / |
| **17** | /* | 20 | / |
| **18** | /* | / | / |
| **19** | /* | 18 | / |
| **20** | /* | 20 | / |
| **21** | /* | / | / |
| **22** | /* | 20 | / |
| **23** | /* | 20 | / |
| **24** | / | 20 | / |
| **25** | /* | 27 | / |
| **26** | /* | 18 | / |
| **27** | /* | 22 | / |
| / - Without the zone of inhibition, microorganisms grew evenly everywhere  /*- Without the zone of inhibition, microorganisms grew evenly everywhere with an observed decrease in the number of treated microorganisms | | | |
