# Supplementary figures and images for: Polyphenolic and Chemical Profiles of Honey From the Tara Mountain in Serbia
Source: Front Nutr. 2022 Jun 24;9:941463. doi: 10.3389/fnut.2022.941463 (PMC9263698; doi:10.3389/fnut.2022.941463)

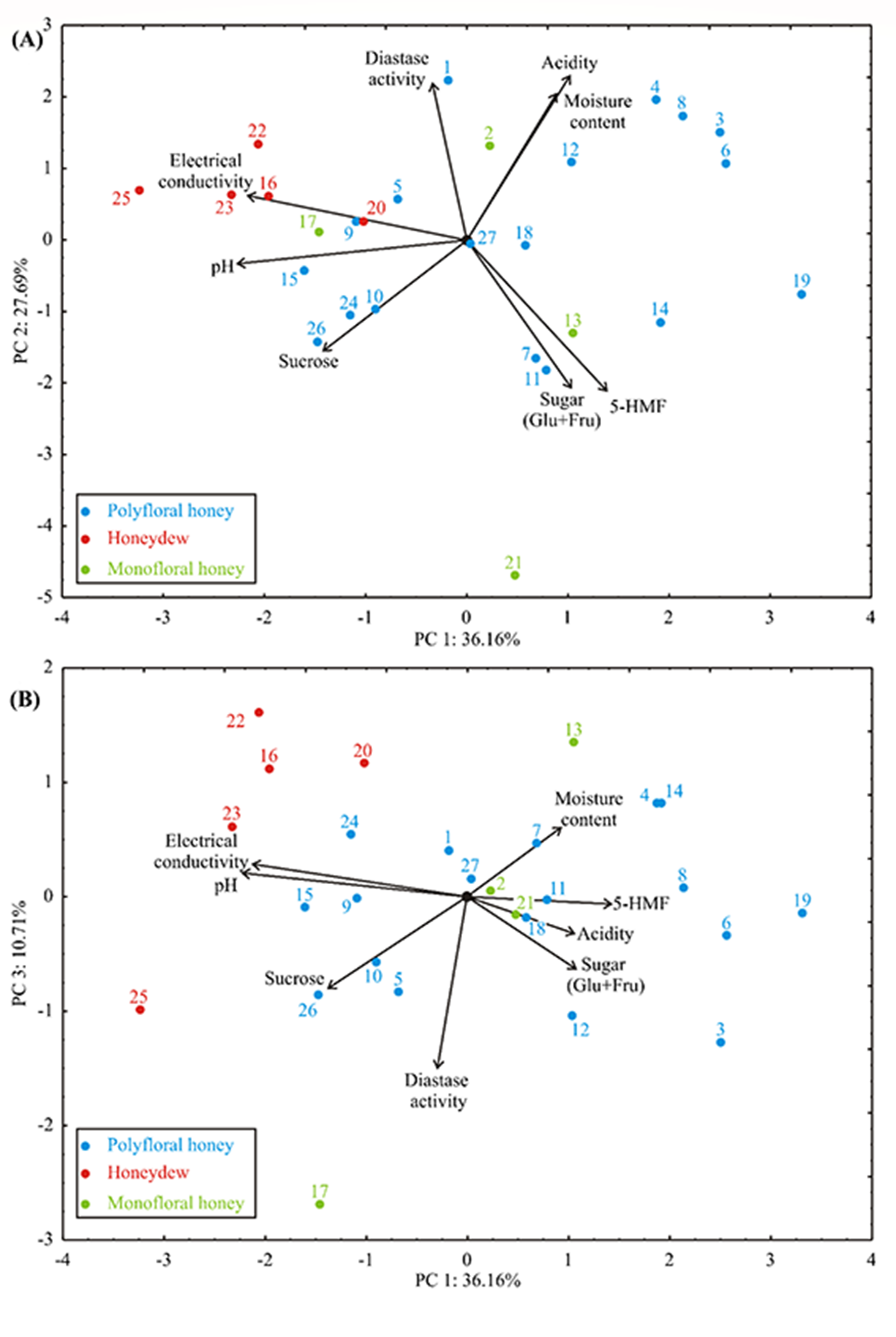

Supplement: Supplementary file 2 [file Image_1.TIFF]

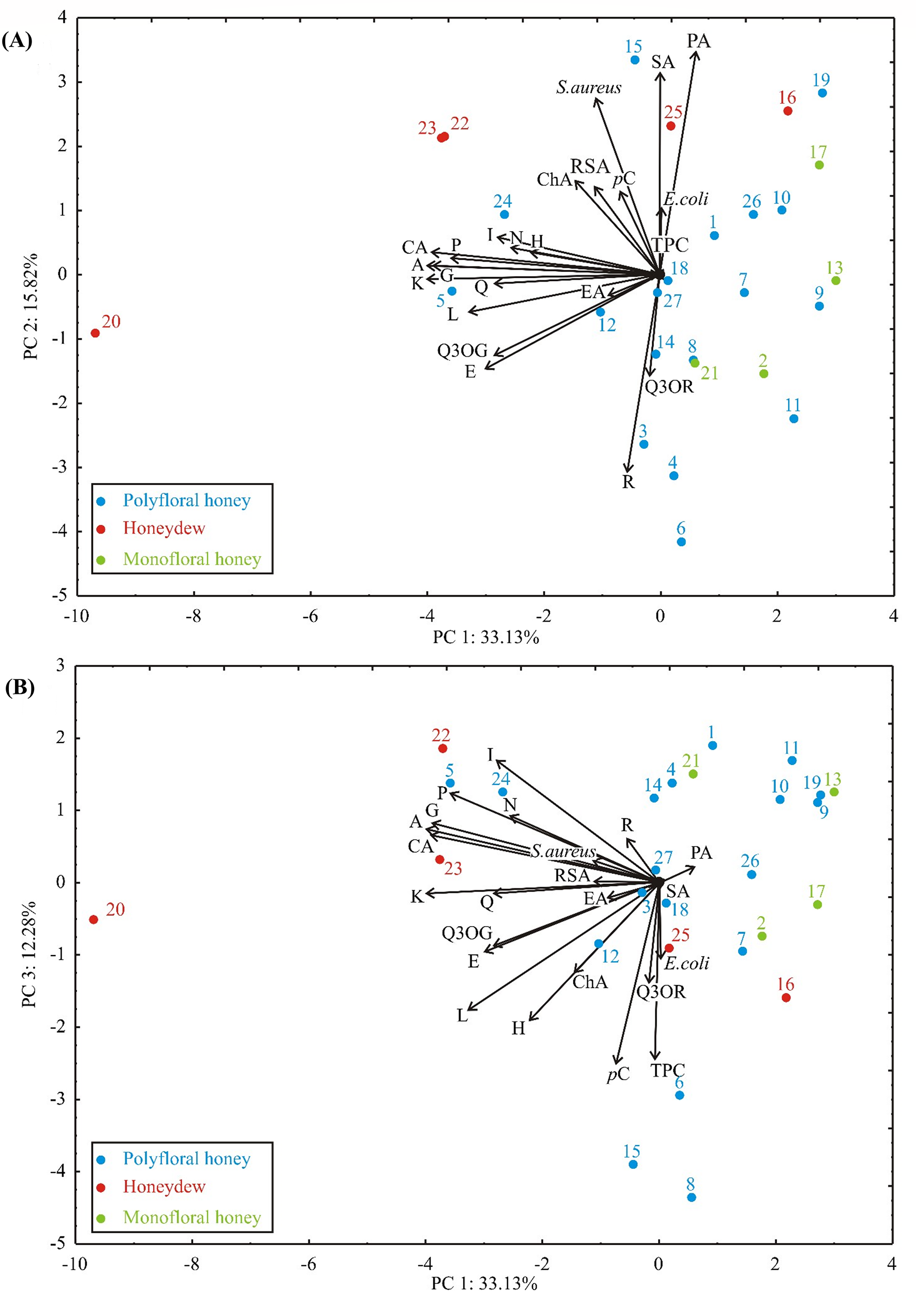

Supplement: Supplementary file 3 [file Image_2.TIFF]
